# How Was Studied the Effect of Manual Wheelchair Configuration on Propulsion Biomechanics: A Systematic Review on Methodologies

**DOI:** 10.3389/fresc.2022.863113

**Published:** 2022-05-02

**Authors:** Capucine Fritsch, Yoann Poulet, Joseph Bascou, Patricia Thoreux, Christophe Sauret

**Affiliations:** ^1^Centre d'Études et de Recherche sur l'Appareillage des Handicapés, Institution Nationale des Invalides, Paris, France; ^2^Arts et Métiers Institute of Technology, Université Sorbonne Paris Nord, IBHGC – Institut de Biomécanique Humane Georges Charpak, HESAM Université, 151 Bd de l'Hôpital, Paris, France; ^3^Hôpital Hôtel-Dieu, AP-HP, Paris, France; ^4^Université Sorbonne Paris Nord, Arts et Métiers Institute of Technology, IBHGC – Institut de Biomécanique Humane Georges Charpak, HESAM Université, 151 Bd de l'Hôpital, Paris, France

**Keywords:** manual wheelchair, configuration, settings, methodology, experiment, kinematics, kinetics, PRISMA

## Abstract

**Background:**

For both sports and everyday use, finding the optimal manual wheelchair (MWC) configuration can improve a user's propulsion biomechanics. Many studies have already investigated the effect of changes in MWC configuration but comparing their results is challenging due to the differences in experimental methodologies between articles.

**Purpose:**

The present systematic review aims at offering an in-depth analysis of the methodologies used to study the impact of MWC configuration on propulsion biomechanics, and ultimately providing the community with recommendations for future research.

**Methods:**

The reviewing process followed the Preferred Reporting Items for Systematic Reviews and Meta-Analyses (PRISMA) flowchart on two databases (Scopus and PubMed) in March 2022.

**Results:**

Forty-five articles were included, and the results highlighted the multiplicity of methodologies regarding different experimental aspects, including propulsion environment, experimental task, or measurement systems, for example. More importantly, descriptions of MWC configurations and their modifications differed significantly between studies and led to a lack of critical information in many cases.

**Discussion:**

Studying the effect of MWC configuration on propulsion requires recommendations that must be clarified: (1) the formalism chosen to describe MWC configuration (absolute or relative) should be consistent with the type of study conducted and should be documented enough to allow for switching to the other formalism; (2) the tested MWC characteristics and initial configuration, allowing the reproduction or comparison in future studies, should be properly reported; (3) the bias induced by the experimental situation on the measured data must be considered when drawing conclusions and therefore experimental conditions such as propulsion speed or the effect of the instrumentation should be reported.

**Conclusion:**

Overall, future studies will need standardization to be able to follow the listed recommendations, both to describe MWC configuration and mechanical properties in a clear way and to choose the experimental conditions best suited to their objectives.

## Introduction

Manual wheelchairs (MWC) allow disabled people with impaired lower limb function to regain autonomy and physical mobility. However, if the MWC is not properly adapted to its user, propulsion can become exhausting, and could favor the appearance of musculoskeletal disorders either through an increase in shoulder net joint moment (1), a decrease in mechanical efficiency (2), or through ranges of motion closer to articular limits (3). Therefore, providing the user with an optimally fitted MWC is crucial and requires in-depth studies of the effect of MWC characteristics on propulsion. Among the different characteristics of a MWC, it is possible to distinguish dimensional (e.g., seat and backrest width and depth) and positional (e.g., camber, backrest, and seat angles) characteristics defining the MWC configuration (i.e., its geometry); and the resulting mechanical properties (e.g., mass, position of the center of mass (CoM), or rolling resistance, etc.). Sometimes, the literature also refers to the word “settings” which is used in the present article as the selected value for a given dimensional or positional characteristic. Besides, various scientific approaches can be implemented to study the effect of MWC characteristics on propulsion, such as physiology, biomechanics, and even human and social sciences. Among the different approaches, biomechanics (i.e., kinematics and kinetics) is particularly well-suited because it relies on physical quantities and measurement systems allowing to obtain instantaneous values, unlike physiological measurements (4).

Tackling the issue of identifying the optimal MWC configuration is challenging because the numerous characteristics involved and the multiple tasks that constitute MWC propulsion (e.g., slope, cross-slope, turning, curbs etc.) result in too many conditions to be tested by a single subject. Hence, researchers tend to isolate a single MWC characteristic in their studies and to focus on one task, generally straight-line propulsion on flat ground. To date, numerous articles have already attempted to quantify the effect of a MWC characteristic on propulsion biomechanics, with a growing interest over the past 20 years. This has led to some authors providing literature reviews with special emphasis on daily (5) or sport displacements (6–8). If some trends could be drawn, contradictory results were also obtained, which could be attributed to differences in methodologies and lack of standardization (7). Indeed, comparing the results of various studies requires dealing with similar experimental conditions (i.e., power output, speed, or metabolic power) (9); and similar MWC configurations to ensure the results portability. Also, some reserves could be expressed on the different studies due to the difficulty in isolating a change in a single setting (10) and by the effective control of the power output across the different tested conditions (11), in particular due to changes in rolling resistance (12, 13), and also due to the alteration of MWC stability (14–16).

Given the diversity of methodologies highlighted by previous authors, the purpose of this systematic review is to identify and report the multiplicity of methodologies used by the literature while studying the effect of MWC configuration on propulsion biomechanics, both for sports and everyday uses, with particular emphasis on experimental task, experimental environment, propulsion speed, MWC configuration reporting, number of configuration under study, measurement systems, MWC used, and participants. Lastly, the discussion strives to provide readers with guidance regarding experiments to be performed, literature analysis, and suggestions for future works.

## Methods

The present review aimed at identifying and analyzing the methodologies used in studies that dealt with MWC configurations and their impact on propulsion biomechanics. The review was conducted following the Preferred Reporting Items for Systematic Reviews and Meta-Analyses (PRISMA) 2020 updated guidance (17). PubMed and Scopus databases were individually searched for relevant articles, regardless of their publication date. The request, initially emitted on December 2020 and updated on March 2022 to add the articles that were published since, focused on retrieving all the articles considering the impact of MWC configuration on propulsion biomechanics and was worded as follows:

(wheelchair[Title]) AND ((setting^*^[Title/Abstract]) OR (configuration^*^[Title/Abstract]) OR (design[Title]) OR (propert^*^[Title/Abstract]) OR (characteri^*^[Title/Abstract]) OR (seat[Title/Abstract]) OR (backrest[Title/Abstract]) OR (camber[Title/Abstract]) OR (wheel[Title/Abstract]) OR (pushrim[Title/Abstract]) OR (handrim[Title/Abstract]) OR (footrest[Title/Abstract]) OR (fork[Title/Abstract]) OR (caster[Title/Abstract]) OR (interface[Title/Abstract]) OR (gear[Title/Abstract]) OR (profile[Title/Abstract]) OR (form[Title/Abstract]) OR (tube[Title/Abstract])) NOT (electric^*^[Title]).

Two authors took part in the screening process (Y. P., C. F.), following the PRISMA flowchart and independently managing half of the records. After duplicate removal, the remaining articles were first screened by title, then by abstract, and finally by full text. Inclusion criterion was studies covering the effect of MWC configuration on propulsion biomechanics from an experimental point of view. In contrast, exclusion criteria were the following: articles involving electric or power assisted wheelchairs; articles about sit-to-stand, reclining, stair-climbing and children-sized wheelchairs; articles involving other propulsion system than manual handrim or no propulsion at all; articles only studying physiological parameters; and articles that were not original studies or not written in English. When in doubt, records were identified and kept in a separate list so that the two authors could reach an agreement. Ultimately, 45 articles were retained and were sorted in main categories according to the characteristics they focused on. For the analysis, the same two authors collected the methodology described in each article, with special attention given to the description of the wheelchair configuration, the subjects' wheelchair experience, and the experimental tasks and devices.

The PRISMA Checklist is appended to this article (Supplementary Material).

## Results

**Figure 1:**
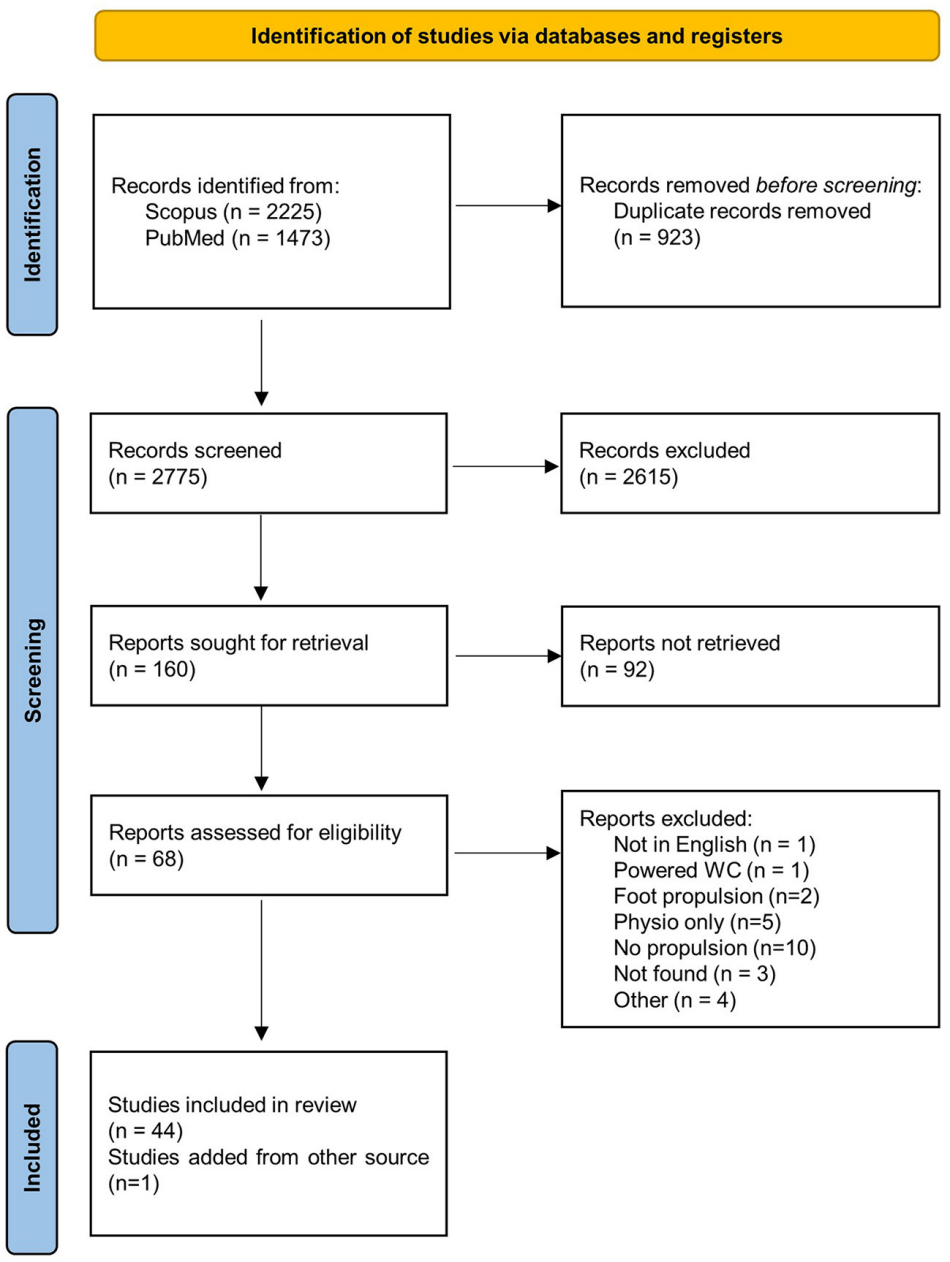
PRISMA 2020 flow diagram.

The compilation on both databases resulted in 3,698 references. After duplicate removal, 2,775 references remained. The screening through the title filter resulted in 160 references. After reading the abstracts, 68 articles were selected, and finally, 44 articles were considered after the full texts were read. An additional relevant article, not identified through the screening process but found in the bibliography of another article, was included in this review (6). This approach is summarized in Figure 1.

The table which led to the redaction of the results section is appended to this article (Supplementary Material).

### General Results

Over the 45 articles remaining from the screening process, 24 articles recruited exclusively MWC users, 17 articles exclusively able-bodied (AB) volunteers, and four articles included both MWC users and AB subjects (Table 1).

**Table 1:** Number and type of participants to each study.

**Number of subjects**	**Able-bodied**		**Spinal cord injury (SCI)**	**Older people**	Athletes^*^
	**Novice**	**Experienced**			
1	(18, 19)				(20)
2–5					(21, 22)
6–10	(23–28) **(****29****) (****30****) (****31****)** (32, 33)		**(****29****) (****30****) (****31****)** (34)		(35–37)
11–20	(38–43) **(****44**)^**^	(45)	(46–49)	(50, 51)	(52–56)
21 +	(57)		(58, 59) **(****44**)^**^	(60)	(61, 62)

*Bold, same study involving different cohorts;*

* *Athletes cohorts described by pathology or classification in the articles, not reported in this table;*

** *Lin and Sprigle (44): cohort of SCI subjects with one subject with ataxia*.

The results were organized per characteristics and gathered into three major parts:

- the characteristics related to the wheels (*n* = 17)
- the characteristics related to seating (*n* = 9)
- the characteristics describing the vertical and horizontal positions of the seat with respect to the rear wheels (*n* =2 0)

In total, three articles studied characteristics included in two of the sections listed above.

### Wheel-Related Articles

Seventeen articles studied wheel-related characteristics focusing either on the rear wheel camber angle (*n* = 8), on the rear wheel (*n* = 3) or handrim (*n* = 3) diameters, on handrim shapes (*n* = 2) or on tire pressure and type (*n* = 1).

#### Camber

Camber is by far the most studied wheel-related characteristic. The 8 articles focusing on camber angle can be organized following their experimental conditions: overground (*n* = 3), on a roller ergometer (*n* = 3) or on a treadmill (*n* = 2).

##### Overground

**Methods:** Three studies investigated the effect of camber angle through overground experiments, involving novice AB subjects (38, 39), or highly trained MWC basketball and tennis athletes (52). As one article is sports oriented and the others focus on daily life displacements, the range of studied camber angles varied noticeably between studies: from 15° to 24° (52), and from 0° to 15° (38, 39). On average, 3 different camber angles (minimum 2, maximum 4) were tested per study. Experiments were carried out using the same MWC without adjustment to the participant (38, 39) or using the same MWC but with seat height adjusted to the athlete's personal MWC by copying their elbow angle when hands were at the handrim top dead center (52).

Wheelchair configurations were described with varying levels of detail among the articles. MWC brand and model were always provided, along with rear wheel, caster and handrim diameters, or seat width, depth, and height.

All the articles specified taking care of preventing “toe-in toe-out” (i.e., alignment of the wheels in the transverse plane) for each camber angle condition. The impact of varying camber on the MWC configuration was controlled and standardized by maintaining the top-to-top rear wheel width constant either at 48 cm (52), or at 40% of the user's arm span (38).

Experiments consisted of a straight-line propulsion over 4-meters long displacements (with 3–4 propulsion cycles before and after the measurement area) at 1 m/s (controlled by measuring the time to complete the 4 meters) (38, 39), and of a combination of a 20 m sprint, linear mobility, and maneuverability drills at maximal speed (52).

**Materials:** Experiments were monitored with motion-capture systems (60 Hz) and 6-components wheel dynamometers (hereafter referred to as “instrumented wheels”) (38, 39), using force plates to compute friction coefficients (39). Others implemented the velocometer device (63) to assess MWC speed (52).

**Parameters of interest:** Parameters of interest included spatiotemporal parameters (time to perform the task, MWC mean and peak velocities, number or frequency of pushes, start, release, and total push angles, stroke patterns, acceleration over the first 2 or 3 pushes) (*n* = 2), kinematics (trunk, shoulder, elbow and wrist peak joint angle and ranges of motion (RoM)) (*n* = 1) and kinetics (maximum power output, external mechanical work, mean rolling resistance coefficient) (*n* = 1).

**Results:** Results showed that the time required to perform a task increased with increasing the camber angle from 15 to 24°, leading to a deterioration of overground sprint and mobility drills performances (52). Similarly, the release angle and the trunk RoM were shown to increase with increasing camber angle (38). The last authors also found a trend of change in the stroke pattern toward a single looping over propulsion (SLOP) pattern (64). Finally, both rolling resistance and total power output were also found to increase with camber (39), explained by the modification of the wheel-ground contact surface.

##### Roller Ergometer

**Methods:** Three studies used commercially available roller ergometers and were all sports oriented (21, 35, 36). They involved experienced MWC users such as MWC basketball and rugby athletes. Camber varied from 9° to 22°, with 3 different camber angles per study. Experiments were systematically performed using the same MWC for all subjects.

The initial MWC configuration was defined through brand, model, weight, overall length, seat and backrest angles, backrest height, seat depth and width, rear-wheel diameter and tire pressure in two studies (35, 36), whereas it was limited to weight, rear-wheel diameter, seat depth and height in the remaining article (21).

One study adjusted the initial configuration to every subject by mimicking the participants' own MWC configuration (21), but the resulting configurations were not reported. For the two other articles, the top-to-top rear wheel width was maintained constant (48 cm) between configurations (35, 36). Finally, two out of three articles specified that a special care was taken to avoid “toe-in toe-out” between configurations.

For all studies, participants were asked to propel at maximal speed for 8 s.

**Parameters of interest:** Parameters of interest included spatiotemporal (MWC average speed, push time, recovery time) (*n* = 2) and kinetic (residual torque, power output) (*n* =2) parameters.

**Results:** Temporal parameters showed conflicting results; one article reported an increase of the push time with camber angle (35) whereas another one did not report any change (21). Higher camber angles were associated with a decrease of the recovery time (21) and of the MWC speed (35). This last result can be explained by the increase of rolling resistance (35, 36) due to the type of contact between the wheels and the rollers. Finally, Faupin et al. (36) showed that reorienting the rollers perpendicular to the wheel plane allowed for a more realistic setup by maintaining residual torque closer to overground or treadmill conditions.

##### Treadmill

**Methods:** Two articles resorted to treadmill experiments to investigate the effect of camber on propulsion. One article studied daily life speeds and involved AB subjects (23), whereas the other article was sports oriented and recruited highly trained basketball and tennis wheelchair athletes (53). Camber angles were between 0° and 9° for the first study and between 15° and 24° for the second one. In each study, the same MWC was used for all participants.

Both articles described the initial MWC configuration through brand, model, weight, handrim or rear wheel diameter, tire brand or pressure and seat height. Seat height was adapted to the participants through the elbow angle when subjects were seated in the MWC with their back resting on the backrest and their hands placed at the handrim top dead center. For Veeger et al. (23), the elbow angle was fixed at 120° for all participants whereas Mason et al. (53) reproduced the angle of participants sitting in their own MWC. When varying configurations, Mason et al. (53) specified maintaining the participant's elbow angle constant and a 48 cm top-to-top rear wheel width across every tested configuration. Mason et al. also specified that “toe-in toe-out” was controlled between configurations, while Veeger et al. (23) took special care in maintaining an equal rolling resistance between configurations.

Both experiments were performed on the same commercially available motor-driven treadmill. For each configuration, subjects were asked to propel for 12 min with increasing speed every 3 min (0.56, 0.83, 1.11, 1.39 m/s) (23), or to propel for 4 min at 2.2 m/s on a 0.7% gradient slope (53).

**Materials:** Both experiments were monitored with video cameras coupled with an optoelectronic motion capture system (53) and EMG electrodes (23).

**Parameters of interest:** Parameters of interest included spatiotemporal parameters (contact and release angles, push and recovery times) (*n* = 2), kinematics (shoulder flexion/extension and abduction/adduction, elbow and trunk flexion, shoulder, elbow, and wrist angular velocities) (*n* = 2), kinetics (rolling resistance, power output) (*n* = 2) and muscular activity (upper limb and trunk muscles activation) (*n* = 1).

**Results:** One study reported differences in push time, push angle and shoulder abduction between 3° and 6° camber angles (23) whereas the other study did not report such results but an increase in shoulder, elbow and trunk RoM in the sagittal plane (53). Finally, one study reported a decrease in rolling resistance with camber (23) whereas the other study found an increase (53).

#### Rear-Wheel Diameter

**Methods:** Rear wheel diameter was studied in three articles involving experienced MWC basketball athletes. Experiments were either performed on the athlete's personal MWC (61), whose configuration was not reported, or on a MWC provided by the authors which was the same for all subjects (54, 55). In that case, the brand, model, gear ratio (i.e., handrim radius divided by wheel radius), camber, and tire pressure were reported. Furthermore, seat height was adapted to each participant through the reproduction of the elbow angle they have in their personal basketball MWC. This elbow angle and the gear ratio were maintained constant for every rear wheel diameter. The authors reported that they were not able to maintain the top-to-top rear wheel width constant between configurations due to camber. Besides, the authors did not report if the change in rear wheel size was associated with an adaptation of both the seat angle and the inclination of the caster fork axle with respect to the MWC frame, while the latter is crucial for turning maneuvers.

Regarding the experimental conditions, one study was carried out on a treadmill at 2.2 m/s (55) whereas the other two studies consisted of overground mobility tests performed at maximal speed such as a 20 m sprint, a linear mobility drill requiring multiple successive forward and backward propulsions and an agility drill composed of sharp turns (54) or the Wheelchair Mobility Performance test composed of 15 mobility exercises such as a 12-meter sprint and a rotation, with and without handling a ball (61).

**Materials:** Measurements involved video cameras (55, 61), a velocometer (54), and an instrumented wheel with additional weight around the hub of the opposing wheel to counterbalance its weight and inertia (55).

**Parameters of interest:** Parameters of interest included spatiotemporal parameters (time to perform the task, stroke frequency, push time, push angle, acceleration over 2 and 3 pushes, peak velocity) (*n* = 3), upper limb kinematics (joints angular displacement at contact and release instants: shoulder flexion and abduction, elbow and trunk flexion, wrist extension) (*n* = 1) and handrim kinetics (resultant and tangential forces, fraction of effective force (FEF), mechanical work, and power) (*n* = 1).

**Results:** Results showed that larger rear-wheel diameter improved sprinting performances without negatively influencing initial acceleration or maneuverability performances (55, 61). If push time, stroke frequency and upper body joint kinematics were not found to be altered by rear wheel diameter, push angle was reported to increase with wheel diameter (55). Larger rear wheel diameters were also associated with smaller handrim total force and larger tangential component.

#### Handrim Diameter

**Methods and Materials:** Three studies focused on the effect of handrim diameter, either involving novice AB subjects using the same MWC (24, 25) or focusing on a single wheelchair racing athlete in his personal racing MWC (20). Handrim diameters ranging from 34 to 37 cm were studied for the racing MWC, while larger handrim diameters, from 32 to 54 cm, were studied in the two other articles (24, 25). In all studies, the handrim diameter varied while keeping the rear wheel diameter constant, inducing an alteration of gear ratio for each configuration.

Description of the MWC characteristics was done through rear wheel diameter and seat depth, width, and height with respect to the ground for one study (25), whereas only the brand and model was given for another article (20) and no information at all were reported in the third one (24).

The experiments on racing MWC took place on a 400 m long athletics track, on which the subject performed laps and 5 min bouts of propulsion, both with 200 m head starts at speeds ranging between 12 and 24 km/h (20). A 500 Hz camera mounted on the MWC allowed for the definition of the propulsion cycle parameters. Participants to the two other studies were asked to propel overground at self-selected speeds over 25 m (25) or for 5 propulsion cycles (24) in a motion-capture equipped runway. One study used pressure sensors, placed inside gloves (25), and the other used an instrumented wheel (24).

**Parameters of interest:** Parameters of interest included spatiotemporal parameters (stroke frequency, push time) (*n* = 1), upper limb kinematics (shoulder and elbow flexion/extension RoM, shoulder adduction/abduction and rotation RoM) (*n* = 1), and upper limb kinetics (hand pressure, mechanical power, power flow) (*n* = 2).

**Results:** Results on spatiotemporal parameters in racing MWC showed that smaller handrims resulted in longer push time and lower push frequency (20). In standard overground propulsion, even if the speed was self-selected, no differences in speed were observed between the different handrim diameters. However, larger handrim diameter was found to be associated with larger shoulder and elbow RoM and larger hand contact forces and pressure (25) and related to greater work and total mechanical energy in upper extremity segments during propulsion (24).

#### Handrim Shape

**Methods:** Two articles studied handrim shape, involving novice AB subjects and using the same MWC for all subjects (26, 57). The articles compared the use of conventional 18- and 20-mm diameter cylindrical metallic handrim either to an oval shape section handrim or to an ergonomically shaped handrim.

The first study (26) used a custom built simulator described by handrim diameter, camber, backrest height and seat height and angle, asking participants to perform two submaximal exercice tests. For the second article, each handrim was mounted on a separate set of wheels with different tire type and pressure, which were reported along with backrest height and seat width and depth to describe the MWC configuration. The experiments consisted of 8-shape displacements at comfort speed.

**Materials:** Measurements were carried out using the custom-built simulator or a Grip VersaTek Wireless System placed inside gloves allowing for the measurement of hand pressure (57).

**Parameters of interest:** Parameters of interest included spatiotemporal parameters (cycle time, push frequency, push angle) (*n* = 1), handrim kinetics (*n* = 1) and upper limb kinetics (hand pressure) (*n* = 1).

**Results:** No significant effect of handrim shape was observed on spatiotemporal propulsion parameters or in power output (26). The contoured handrim design was related to reduced levels of contact pressure on most hand regions, however it concentrated a high level of pressure on the medial phalanges, preventing the authors from recommending this feature (57).

#### Tire Type and Pressure

**Methods:** One article focused on the effect of tire type and pressure on physical strain and propulsion technique (40). Experiments were conducted on novice AB subjects. Two tire types (pneumatic and solid) and four pneumatic tire pressure conditions [100, 75, 50, and 25% of the recommended pressure (i.e., 6 bars in that case)] were evaluated. A configuration with extra mass (5 and 10 kg) added on the rear wheel axle was examined both for 100% pressure pneumatic tires and for solid tires. All the participants used the same MWC, defined by brand, model, total mass, rear wheel diameter, camber and seat and backrest angles with respect to the horizontal and frontal planes, respectively. The experiments consisted of 4-min bouts of propulsion at 1.11 m/s on a level treadmill.

**Materials and parameters of interest:** The MWC was equipped with 2 instrumented wheels, allowing for the measurement of spatiotemporal (push time, cycle time, push frequency and push angle) and kinetic (total and tangential forces, propelling torque, FEF and power) parameters.

**Results:** Results showed that lower tire pressure resulted in smaller cycle time and push angle, as well as in significantly lower FEF and higher power output. Solid tires were also found to increase power output. Additional mass did not have a significant impact on propulsion technique (timing and force application) or power output, although a trend of increasing power output with solid tires was observed.

### Seating-Related Articles

Nine articles studied seating-related characteristics, with focus on seat and backrest angles (*n* = 6), backrest height (*n* = 1), and footrest positioning (*n* =2).

#### Seat and Backrest Angles

**Methods:** Six articles focused on seat and backrest angles. Two articles included the effect of both angles on propulsion with elderly people (50, 51) and four articles focused only on the effect of seat angle either on spine curvature and scapular kinematics during propulsion in users with spinal cord injury (SCI) (58), on mobility and propulsion kinematics in elite MWC rugby players (37), on seating ergonomics and mobility efficiency in SCI users (46), or on the position of the MWC-user's CoM during propulsion of one AB user (18). Overall, seat angles were studied in the range of 0° to 14°; and backrest angles were studied between 95° and 105°. All articles defined seat and backrest angles with respect to the horizontal plane. However, one article, using a specific platform, tilted the entire MWC during its experiments (18). Except for the characteristics of interest (i.e., seat and backrest angles), the descriptions of the MWC configurations were scarce in all the retrieved studies.

Experiments were conducted at comfort speed (~1 m/s) using a custom-built ergometer (18, 50, 51), the participants' own MWC on a roller ergometer (58), or the same MWC for all participants, either using the same predefined configuration for all participants on a treadmill (46) or mimicking each participant's own MWC configuration during maximal speed overground mobility tests (37).

**Materials:** Experiments were monitored with optoelectronic motion capture systems (50, 51, 58), video cameras (37, 46), or inertial measurement units (IMU) (37). An instrumented wheel was used in two studies, adjusting the second wheel inertial properties by adding weight to it (50, 51).

**Parameters of interest:** Measurements included spatiotemporal data (push frequency, contact, release and total push angles, time to perform the task) (*n* = 4), kinematics (shoulder rotations, global CoM displacement) (*n* = 2) and kinetics (handrim forces, FEF, shoulder net joint moments, power output, mechanical efficiency) (*n* = 3).

**Results:** Results showed that push angle increased with increasing seat (46, 51) and backrest angles (51). During sprint and agility tasks, reduced seat angle was found to reduce the time required to perform the task (37). Regarding kinematics, seat angle did not alter glenohumeral rotation, but higher inclination resulted in higher scapulothoracic internal rotation (58). Regarding kinetics, FEF was improved with increasing seat and backrest angles (50) without affecting peak and mean shoulder net joint moment in elderly people (51).

#### Backrest Height

**Methods:** One article focused on backrest height (59). Experiments were conducted in SCI users with injury from T8 to L5 vertebrae. Two backrest heights were tested, described as a fixed height of 40.6 cm for the highest condition and subject-specific (50% of the user's trunk length) for the lowest. All the participants used an identical MWC, provided into two seat widths to accommodate various body sizes but the MWCs characteristics were not reported. Only backrest height varied between tested configurations. The experiment consisted of four 30-s propulsions at 0.9 m/s on a treadmill, with varying slope inclination (0–3°).

**Materials and parameters of interest:** An optoelectronic motion capture system was used, coupled with two instrumented wheels allowing for the determination of spatiotemporal (push time and push angle), kinematic (shoulder peak extension and shoulder flexion/extension RoM), and kinetic (mechanical effective force) parameters.

**Results:** Results showed a smaller push time and push angle and a higher push frequency with the higher backrest, which also resulted in smaller shoulder extension angles at the beginning of push phase and smaller shoulder flexion/extension RoM. The mechanical effective force was not found to be altered by seat height.

#### Footrest Positioning

**Methods:** Two articles studied the impact of footrest positioning on MWC propulsion in AB participants. One examined the effect of footrest angle, defined through knee flexion, on MWC turning maneuver (27), while the second studied the effect of footrest height, defined through hip flexion, on MWC linear acceleration during a straight-line displacement (45). The first article examined fully extended (0° knee flexion) and fully flexed (120° knee flexion) positions during angular velocity tests, requiring the participants to rotate the MWC over 900° (2.5 full turns) as fast as possible (27). Prior to angular velocity tests, the MWC-user's CoM, overall length, rolling and turning resistances and yaw mass moment of inertia (MoI) were determined. For the second article, three hip flexion angles (0°, 45°, and 90°) were tested, and the participants were asked to propel at maximal speed on a custom-built roller ergometer for 20 s.

Both articles used the same MWC for all participants without adjustments. The MWC characteristics were described through brand, model, seat width and depth, backrest height and rear wheel diameter for both articles, plus seat angle and rear wheel camber (45) or rear-wheel axle plate position, MWC-user's CoM, overall length, rolling and turning resistances and yaw mass MoI (27). During the experiments, the modifications in footrest positioning (height or angle) did not impact any other MWC geometrical characteristics.

**Materials and parameters of interest:** Both experiments were monitored by video cameras, allowing for the determination of spatiotemporal (task time, peak velocity and acceleration during the first 2 s from standstill, covered distance after 1, 2, and 3 s from standstill) (*n* = 2), and kinematic (trunk flexion/extension positions) (*n* = 1) parameters.

**Results:** Results showed that fully flexed knee position resulted in a greater angular velocity, a more rearward position of the CoM and thus a decrease in rolling and turning resistances. Results also showed an improvement of the covered distance during the first 3 s when thighs were parallel to the floor and a reduced capacity to accelerate was noted with hip completely flexed (thighs on the trunk with vertical trunk). Regarding trunk kinematics, flexion/extension average position was altered but the trunk only actively participated in the first push in the condition with thighs parallel to the floor.

### Seat Vertical and Horizontal Positions

Twenty articles studied the position of the seat relative to the wheels, with focus on the vertical position (seat height) (*n* = 6), on the horizontal position (seat fore-aft position) (*n* = 6), or on both (*n* = 8).

#### Seat Vertical Position (Seat Height)

The 14 articles studying seat height can be organized following their experimental propulsion conditions: overground (*n* = 5), on a treadmill (*n* = 2), on a roller ergometer (*n* = 4) or on a stationary wheelchair simulator (*n* = 3).

##### Overground

**Methods:** Articles studying the effect of seat height on the biomechanics of overground propulsion involved either AB participants (41) or experienced MWC users (37, 47, 56, 62). Three articles were sports oriented: two focused on MWC basketball (56, 62) and one on MWC rugby (37). For these studies, participants either performed the Wheelchair Mobility Performance test gathering 15 sport-specific tasks (56, 62) or a combination of 5 m sprints, Illinois Agility Test, and a specific “skill” test (37). Other experiments consisted of overground propulsion at a self-selected speed (47) or of maximal speed propulsion over a 3 m long ramp with a 1:12 slope (41). Two articles (56, 62) used the athlete's own MWC and moved the seat up and down by 7.5% of its initial position. Two other studies used a custom-made adjustable MWC and modified seat height either by plus and minus 15 mm (37) or using four pre-selected heights covering 10 cm from the lowest to the highest position (47). The last article tested four seat heights defined from elbow flexion angle (0°, 30°, 60°, and 90°) using the same MWC for all participants (41).

MWC configurations were either not described (41, 56, 62) or described through seat depth, angle, and tire pressure (37) or through brand, model, rear and front wheel diameter and type, handrims diameter, seat width and depth and camber angle (47).

While varying seat position, one article specified keeping constant “all other configuration parameters” (37), and two other studies mentioned “preserving other chair ratios” and modifying backrest and footplate heights by the same amount as seat height (56, 62). One article provided the seat angles associated with the highest and lowest tested seat heights (47).

**Materials:** Experiments were monitored using an optoelectronic motion capture system combined with instrumented wheels (47), EMG electrodes (41), IMUs (37, 56) and video cameras (37, 62).

**Parameters of interest:** Parameters of interest included spatiotemporal parameters (time to perform the task, push frequency, push and recovery times, distance traveled per stroke, contact, release, and total push angles, MWC peak or average forward and rotational speed and acceleration) (*n* = 4), kinematics (elbow flexion angle) (*n* = 1), kinetics (axial, tangential and radial handrim forces, FEF, peak propelling torque) (*n* = 1) and muscular activity of the upper limbs (*n* = 1).

**Results:** Results showed an increase of both push time and push angle with lower seat heights (47). Regarding task time, contradictory results were obtained with either a decrease (56, 62) or an increase (37) with lower seat positions. Increasing seat height was also found to decrease the elbow flexion angle when the hand is at the handrim top dead center (47). Regarding handrim forces, lower seat positions were found to increase peak radial and axial forces but were not found to impact the tangential component, the mean FEF or the peak propelling torque (47). Finally, higher activation levels of the pectoralis major and of the triceps muscles were associated with lower seat positions (41).

##### Treadmill

**Methods:** Two articles used treadmill experiments, involving AB subjects propelling for 12 min with increasing speed every 3 min (0.56, 0.83, 1.11, 1.39 m/s) (28) or SCI MWC users propelling for 6 min at 1 m/s (46).

The first study (28) used the same solid-frame basketball MWC for all participants, with an adapted wood seat allowing for seat height modifications independently from seat fore-aft position. The MWC initial configuration was described through brand, weight, caster and rear wheel diameters, handrim diameter, tire pressure and camber angle; and four different seat heights were investigated, defined through the elbow extension angle when the hand is placed at the handrim top dead center (100°, 120°, 140°, and 160°). The second study (46) also used a single MWC for all participants adapted to fit each subject by modifying seat width and backrest angle. The MWC configuration was described by brand and model and two seating positions were investigated, defined through the seat angle (5° and 12°), with a difference in seat height of 55 mm.

**Materials:** Measurements involved video cameras for both articles and EMG electrodes (28).

**Parameters of interest:** Parameters of interest included spatiotemporal parameters (push angle, push frequency, contact and release angles) (*n* = 2), kinematics (trunk elbow and shoulder flexion/extension) (*n* = 1) and muscular activity (left arm and trunk muscles) (*n* = 1).

**Results:** Results showed a decrease in push angle and push frequency with increasing seat height in both articles. Regarding kinematics, higher seat heights resulted in a decrease of elbow flexion and shoulder extension and abduction, while elbow extension and trunk flexion were increased (28). Finally, a shorter activation period was found with a higher seat for upper-limb muscles, except for the triceps, which exhibited a longer activity (28).

##### Roller Ergometer

**Methods:** Over the four articles that used a roller ergometer, two included both AB participants and MWC users for comparison during daily locomotion (29, 30) and two focused on sports MWC, involving experienced MWC athletes. The latter studied rugby with propulsions at maximal speed for the equivalent of a 14 m sprint (21) and racing with propulsions at 60% of the participant's maximal speed for three 90-s trials (22). For daily locomotion, participants were asked to propel at a self-selected speed for 15 propulsion cycles (29, 30).

All the articles used a single adjustable MWC for all their participants. The initial MWC configuration was either described through weight, rear-wheel diameter, seat depth and height (21); through brand, camber, seat and seat-to-backrest angles (22); or not described at all (29, 30). Overall, the configuration was specified to be controlled and/or maintained constant while changing the seat height. Regarding the number of tested configurations, two articles tested three seat heights (44, 47, 50 cm; distances taken between the ground and the back of the seat) (29, 30); one article investigated the participant's usual MWC seat height and two other heights (3 and 6 cm above the usual seat height) (21) and the last article tested two positions which were defined by the seat position at which the user's distal phalanges were aligned with the lowest portion of the handrims and 10 percent of the subject's arm length higher (22).

**Materials:** Experiments were performed on custom-built roller ergometers for all the articles, except for one that used a commercially available ergometer (21). Measurement systems included an optoelectronic motion capture system (29, 30), video cameras (22), and built-in sensors within the ergometer (21). Two articles included EMG electrodes on the participant's upper limbs (22, 29).

**Parameters of interest:** Parameters of interest included spatiotemporal parameters (cycle, push and recovery times, push frequency, push, contact and release angles, mean MWC velocity and push phase acceleration) (*n* = 3), kinematics (trunk, shoulder, elbow and wrist RoM, trunk, arm and hand angular velocities and accelerations, shoulder, elbow and wrist joints velocities) (*n* = 2) and muscular activity of the upper limb muscles (*n* = 2).

**Results:** Results showed a decrease in cycle time (21, 30), an increase in push phase acceleration (21), and a decrease in upper limb RoMs (30) while increasing seat height. Regarding muscle activity, contradictory results were found with seat height associated both with an increase in upper-limb muscle activation during push phase (29), but also with a decrease in muscle activation over the whole cycle, including both push and recovery phases (22).

##### Stationary Wheelchair Simulator

**Methods and materials:** Three articles used stationary simulators to investigate the effect of seat height. Studies involved either AB participants (42), SCI subjects (48) or both (31). Participants were asked to propel at daily life speeds of 3 km/h with power output at 7.5 W for all participants (31), or between 0.42 and 0.83 m/s and with individual power output ranging from 5.5 to 14 W (48), or to perform maximum isometric exercises during 6 s per configuration (42).

The stationary wheelchair simulators were either described through camber, seat and seat-to-backrest angles, wheel and handrim diameters, size of the rim tube, top-to-top rear wheel width and seat fore-aft position (48); through seat fore-aft position and handrim diameter (31), or through handrim radius (42). In one article, the simulator was adapted to every participant by aligning the subject's acromion vertically above the wheel axle (48).

Regarding the investigated seat heights, all three studies tested seat heights defined through the elbow flexion angle when hands are at the handrim top dead centers. One included two seat heights with values of 90° and 100°, corresponding to an average difference between seat heights of 3.3 cm (31). Another compared eight seat heights with steps of 10° from 70° to 140° (48). The last study investigated nine configurations, defined from both shoulder and elbow angles to define both vertical and fore-aft position of the seat, (shoulder: from 30° to 70° with 10° increments; elbow: from 65° to 100° with 5° increments) (42). When altering seat height, one article mentioned keeping all the other settings constant (48). The seat and the wheels were separated in the other two custom-made ergometers (31, 42), allowing for the independent modification of seat height.

**Parameters of interest:** Parameters of interest included spatiotemporal parameters (cycle, push, and recovery times) (*n* = 1), kinematics (trunk, shoulder, and elbow flexion/extension RoMs) (*n* = 1), kinetics (FEF, average and peak propelling torque) (*n* = 1) and upper limb muscles activation (*n* = 1).

**Results:** Despite the small difference (3.3 cm) between the seat heights tested, probably explaining the lack of differences observed in propulsion temporal characteristics (cycle, push, and recovery times), the lower seat position resulted in higher upper-limbs RoM in Hughes et al. (31). Regarding kinetics, FEF was found to increase with seat height (48) contrary to the average and peak torques during isometric measurement (42). Similarly, seat height was not found to influence anterior deltoid and triceps muscles activities (42).

#### Seat Horizontal Position (Seat Fore-aft Position)

Fourteen articles focused on seat fore-aft position and are presented below according to their experimental conditions: overground (*n* = 6), on a roller ergometer (*n* = 6), or on a stationary wheelchair ergometer (*n* = 2).

##### Overground

**Methods:** Six articles used overground propulsion and involved experienced MWC users (37, 47), novice AB participants (19, 32, 43) or older people with no information on their MWC experience (60). One article focused on MWC rugby athletes (37). Participants were asked to propel at a comfortable self-selected speed either for at least 4 cycles on a linear path on 4 different surfaces (60), for 10 m (32), for 20 m (47) or for 30 cycles (43); to perform a combination of 5 m sprints, Illinois agility test and a specific “skill” test (37), or a combination of a 15 m straight line sprint and a slalom course (19).

The six studies used a single MWC for all their participants. The initial configuration was either described through MWC brand, rear-wheel diameter, tire type, handrim diameter, front wheel type, seat width and depth and camber angle (47); through brand, mass, seat width, depth, height and inclination, cushion thickness and type, side guards material, rear and caster wheel types and diameters, handrim material and camber (60); through brand and seat height (32); through fore-aft seat position (19); through seat height, angle, and tire pressure (37) or not described (43). All articles except one (43) used an adjustable MWC, but only three reported a MWC adaptation to each participant (37, 47, 60), and Kotajarvi et al. (47) specified this adaptation to be the reproduction of the participant's backrest and footrest heights.

In all studies, changes in seat fore-aft position were defined by the difference with respect to an initial configuration, but only one study (19) also provided the actual fore-aft position of the seat with respect to the rear wheel center. The total amplitude of variation varied between 3 cm (37) and 8 cm (47, 60). Four studies specified that some or all the other settings were maintained constant and/or controlled between configurations (32, 37, 47, 60).

**Materials:** Regarding measurement devices, the experiments used optoelectronic motion capture systems (32, 47), video cameras (37), instrumented wheels (47, 60), IMUs or accelerometers (37, 43), and EMG electrodes (19, 32).

**Parameters of interest:** The parameters of interest included spatiotemporal parameters (average speed, stroke time and frequency, stroke distance, push and recovery times and contact and release angles) (*n* = 3), kinematics (*n* = 1) (trunk, shoulder, elbow, and wrist ROMs), kinetics (total handrim force and, radial and tangential components, propelling torque, FEF) (*n* = 2) and upper limb muscles activations (*n* = 3).

**Results:** Results showed that the fore-aft seat position did not impact push frequency and stroke distance (60), but a more forward seat position was found to improve skill test performances with sports MWC (37). Regarding kinematics, a forward position of the seat was found to increase the RoM of all the upper limb joints (32). As for kinetics, a rearward seat position was associated with lower total and tangential handrim forces (60) and with reduced upper limb muscle activity in daily life (32) and for sport (19).

##### Roller Ergometer

**Methods:** Six articles used a roller ergometer to study the effect of seat horizontal position, all involving experienced MWC users, and two also including AB participants for comparison (29, 30). Sport propulsion was considered in two articles, with focus on rugby with propulsions at maximal speed for the equivalent of a 14 m sprint (21) and racing with propulsions at 60% of the participant's maximal speed for three 90-s trials (22). For daily life locomotion, participants were asked to propel at self-selected speed for 15 propulsion cycles (29, 30) or at comfort speed for two trials of graded propulsion at 8% incline (34, 49).

Most studies used a single adjustable MWC for all their participants, except for two studies that used two similar adjustable MWCs with different seat width to cover all anthropometric differences (34, 49). Overall, four studies added custom-made adjusting systems on a MWC (29, 30, 34, 49). Three studies used individual initial configurations, either based on the reproduction of the participant's own MWC (21) which were not reported or based on the vertical alignment of the subject's shoulder with the rear-wheel center (34, 49). The initial MWC configuration was either described through weight, rear-wheel diameter, seat depth and height (21); through brand, wheel camber, seat and seat-to-backrest angles (22); through brand, seat width and camber (34); through brand and seat width (49); or not described (29, 30).

Seat fore-aft position was identified by the horizontal distance between the rear-wheel axle and the back of the seat, either as an absolute value (22, 29, 30), or relative to its initial position (21, 34, 49).

Regarding the number of tested configurations, the studies investigated between 2 and 4 fore-aft positions of the seat with the total amplitude of variation going from 6 to 10 cm. Three articles specified controlling and/or maintaining the MWC configuration constant while changing the seat fore-aft position (21, 22, 30).

**Materials:** Regarding experimental conditions, studies were performed on a commercially available ergometer (21), on custom-built roller ergometers (22, 29, 30), or on a roller ergometer with removable flywheels (34, 49). Measurement systems included optoelectronic motion capture systems (29, 30, 34, 49), video cameras (22), instrumented wheels (34, 49), and surface (22, 29) or fine-wire EMG electrodes (34, 49).

**Parameters of interest:** The parameters of interest included spatiotemporal parameters (cycle, push, and recovery times; push frequency; push, contact and release angles; MWC average speed and push phase MWC mean acceleration) (*n* = 5), kinematics (trunk, shoulder, elbow, and wrist RoM; and trunk, arm and hand angular velocities and accelerations) (*n* = 2), kinetics (propelling torque and power; shoulder net joint force) (*n* = 1) and upper limb muscle activation (*n* = 4).

**Results:** Results showed an increase of the push angle (21, 30), an increase of upper limb joint RoM (30), and a decrease of peak elbow extension velocity (22) with a more backward seat position. The net shoulder force direction was also impacted by the seat fore-aft position (49). Finally, EMG outputs showed that the combination of backward and low seat position was associated with the lowest muscle activation level (pectoralis major and anterior deltoid muscles) (22, 34). However, other authors found contradictory results with a higher muscle activation for a backward position of the seat (29).

##### Stationary Wheelchair Simulator

**Methods:** Two articles used stationary MWC simulators (31, 42) to study the effect of seat fore-aft position on propulsion, involving either AB subjects (42) or both AB and SCI subjects (31); either performing maximum isometric pushes (42), or propelling in a straight line at 3 km/h with a power output of 7.5 W (31).

In both articles, the fore-aft position was modified through the position of the handrim hub relative to the back of the seat. The other MWC characteristics were not described. One study aligned the backrest with the hub as an initial position, and then defined two other tested positions with respect to the user's arm length (31). The second article considered 9 positions by altering both the vertical and fore-aft rear wheel positions, defined through shoulder and elbow flexion angles (42).

**Materials:** In addition to the measurements provided by the simulators, rotary potentiometers (31) and EMG electrodes were used (42).

**Parameters of interest:** The parameters of interest were kinematics (upper limb joint RoM) (*n* = 1), kinetics (peak and average torque, force vector) (*n* = 1) and muscular activity (*n* = 1).

**Results:** Results showed greater elbow and shoulder RoM in the frontal and transverse planes for frontward seat positions whereas shoulder RoM in the sagittal plane was greater for rearward seat positions (31). Isometric torque increased for a rearward seat position and the upper limb muscles seemed to be recruited differently between the handrim positions (42).

## Discussion

Numerous articles revolving around MWC configuration and its impact on propulsion biomechanics were published in the last 40 years. From this consideration, the present review aimed at identifying and reporting the multiplicity of methodologies used in the literature to investigate the effect of MWC configurations on propulsion biomechanics, both for sports and everyday use. In doing so, this review highlighted issues in the methods implemented to study MWC configuration that are discussed below.

### Standardizing the Description of MWC Configuration

#### An Intelligible Description of MWC Configuration

The first issue raised by this review is the lack of essential details in the description of MWC configurations despite it being crucial to ensure results portability to clinical or sports fields and to allow the comparison and aggregation of results between studies. Indeed, some articles reported information limited to MWC brand and model, which does not provide information about MWC characteristics. Therefore, it requires the reader to make tedious research on manufacturer commercial and technical booklets, which also limits the comparison of studies. Similarly, reporting tire type does not provide the reader with intelligible information; reporting rolling and steering resistances would be more informative. However, the level of essential details that are required also depends on the experimental propulsion condition (i.e., overground, treadmill, roller ergometers or stationary wheelchair ergometer) and the studied propulsion task. For instance, when studying turning, assessment of both the MWC CoM location and yaw mass MoI are crucial, which is not the case when studying straight line propulsion. Also, reporting the MWC mass when propelling on a roller ergometer is not relevant because the only useful information is the rolling resistance resulting from the load applied by the rear wheels on the rollers.

Hence, it seems necessary to standardize MWC configuration description, which would facilitate the comparison of results between studies and the reproduction of similar experimental conditions. It is also critical to ensure the efficient integration of results to clinical and sports fields for the benefit of MWC users. The following list displays the MWC parameters that should be systematically reported for an intelligible description of the MWC characteristics directly or indirectly linked to the MWC configuration:

- Dimensional parameters: rear wheel, caster and handrim diameters; seat width and depth; backrest width and height; rear and front wheel track; wheelbase; caster trail; footrest length.
- Positional parameters: rear wheel camber; backrest and seat angles; back of the seat fore-aft position with respect to the rear wheel axle; back seat height with respect to the ground; footrest position and orientation; fork axis angle.
- Mechanical parameters: inertial parameters (mass, CoM, yaw mass MoI); rolling and steering resistances.

However, obtaining all those parameters is not straightforward and determining MWC positional, dimensional, and mechanical characteristics is time consuming (12, 13, 65–69). The development of material and computer tools that allow a quick and easy determination of MWC characteristics would also favor their more systematic reporting in future publications. Additionally, reporting all these details will take a noticeable writing space in papers where word limits encourage not to report such level of information. Sharing additional data as Supplementary Material, for instance, would allow to overcome this issue.

#### Description of Configuration Changes

Another challenge is the standardization of how the configuration is altered between tested configurations during the experiments. Firstly, some articles only reported the range of variation in the characteristic of interest without reporting the actual initial setting and the whole description of the MWC configuration, thus preventing reproducing their experiments. Secondly, most of MWC geometrical characteristics are interdependent (70), and one must be careful, when modifying a MWC dimensional or positional characteristic, to consider its impact on the others. Indeed, modifying one characteristic could require several adjustments to keep the rest of the configuration constant (e.g., the modification of rear wheel camber implies a variation in at least nine parameters of the MWC) (10). However, it might be impossible for many commercial MWCs. In that case, impacted settings should be monitored and reported.

Also, there can be an ambiguity between modifying a geometrical characteristic and modifying the mechanical system that allows this change. For instance, altering the seat angle could necessitate several mechanical changes if the seat height is expected to be maintained constant. Most of the articles indicated that the other settings were either “controlled” or “maintained constant,” without providing a clear overview on what was actually unchanged and how it impacted the results.

Therefore, researchers are encouraged to select a MWC with adjustment modalities that do not generate other setting changes than the one under study. When not possible, a careful examination of the interdependent characteristics and how to correct them to maintain the rest of the configuration constant is necessary. When performed, authors are encouraged to specify that they have checked all the characteristics of the MWC for each configuration. Regarding mechanical properties, changes in MWC configuration result in changes in MWC-user's CoM position and yaw mass MoI; and in rolling and turning resistances. The researchers are thus incited either to try to compensate or, at least, to assess their impact on the provided results.

#### “Absolute” vs. “Relative” Formalisms

Beyond the fact that, in the literature, multiple designations can sometimes refer to the same geometrical characteristic (e.g., seat fore-aft position and rear wheel axle fore-aft position), Table 2 illustrates that two formalisms are commonly used to describe MWC configurations and their changes. Firstly, MWC characteristics can be expressed as dimensional measurements such as distances between points or angles between planes, as defined by the international standard ISO 7176-7 or in usual MWC provider datasheet. This formalism will be referred to as “absolute.” Differently, MWC parameters can be defined according to the user's anthropometric parameters, the most frequent example being seat height defined from the user's elbow extension angle when hands are placed at the handrim top dead center (28). This formalism will be referred to as “relative” hereafter.

**Table 2:** List of the different geometrical characteristics of a MWC and definitions used throughout the literature.

**MWC geometrical characteristics**	Representation(s)^*^		**Type**	**Different definitions in literature**
	**Absolute**	**Relative**		
Wheel camber	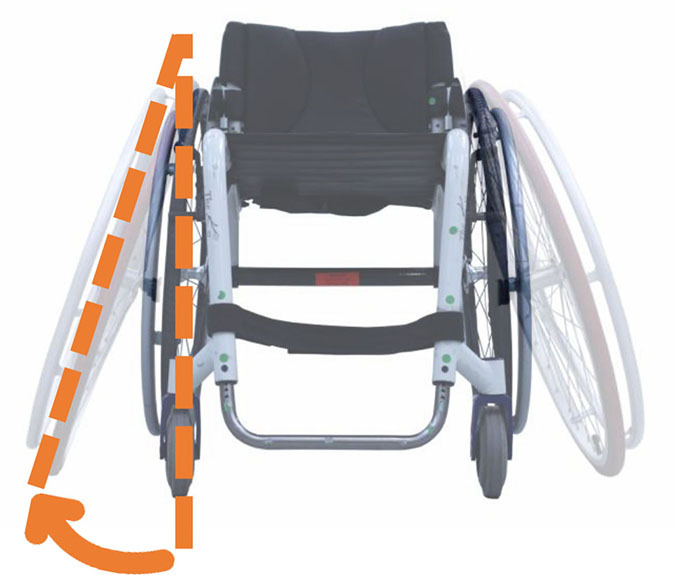		*Positional*	• Angle of the main wheels in relation to the vertical
Wheels/ handrim size	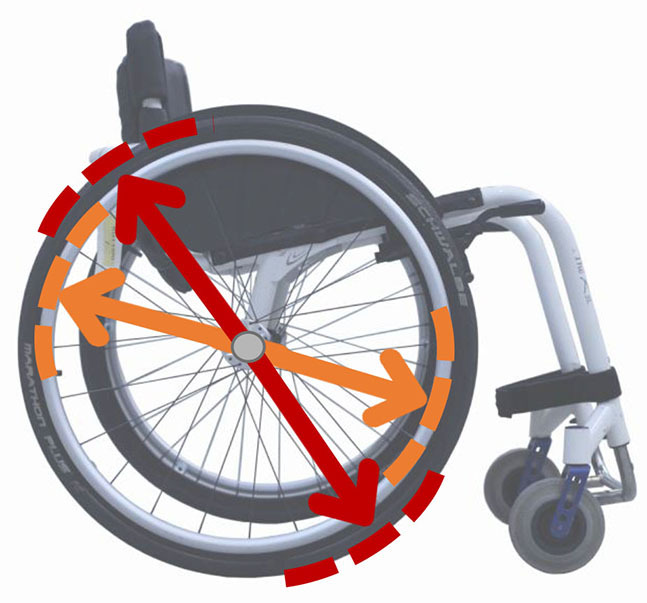		*Dimensional*	• Diameter of the rear-wheels only (Change in the gear ratio) • Diameter of the handrim only (Change in the gear ratio) • Diameter of both the rear-wheels and the handrim (No change in the gear ratio)
Seat angle	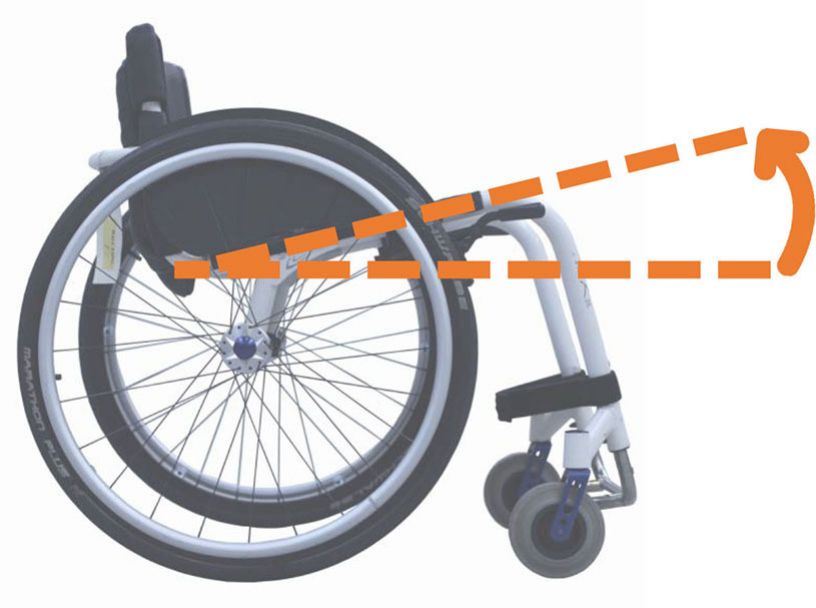		*Positional*	• Seat angle from the horizontal plane • System tilt angle (seat and backrest tilt) • Seat dump
Backrest angle	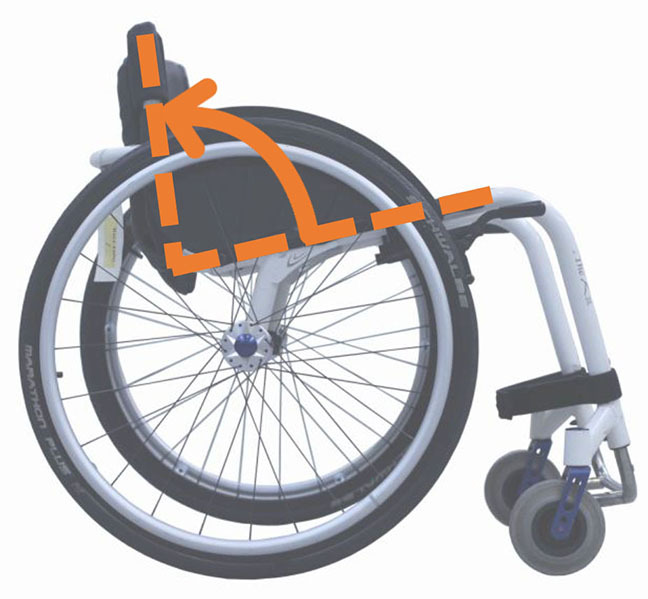		*Positional*	• Seat-to-backrest angle • Angle between backrest and the horizontal or vertical plane
Backrest height	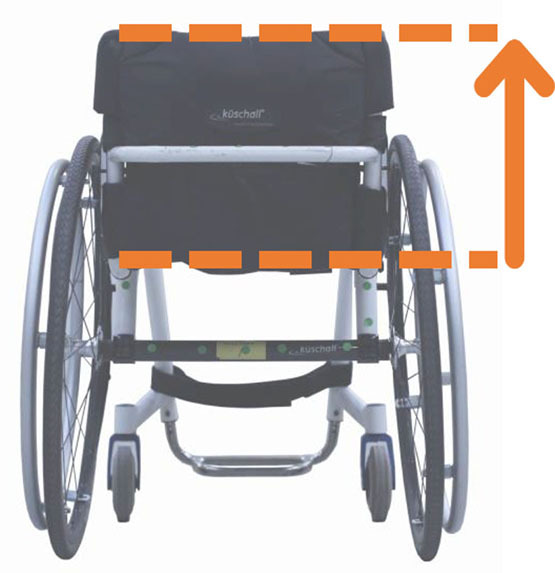	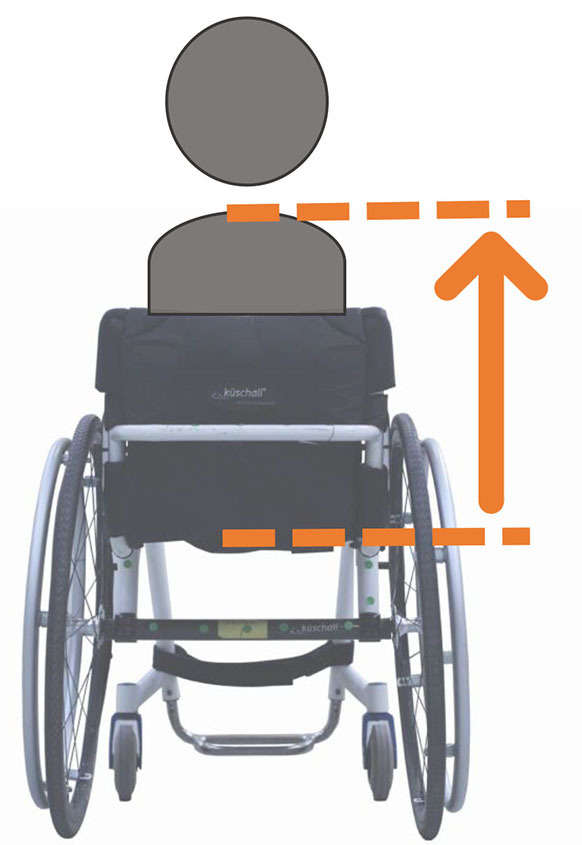	*Dimensional*	• Distance between the back of the seat and the top of the backrest • Backrest placed at a specific trunk height
Footrest positioning	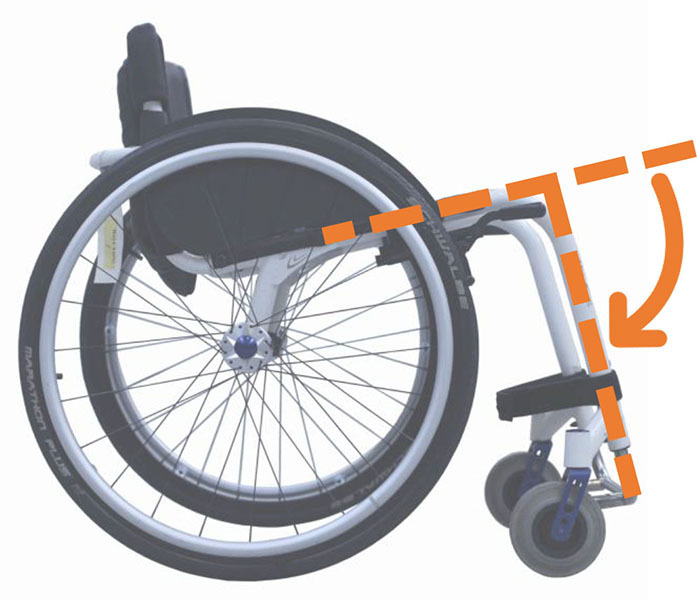	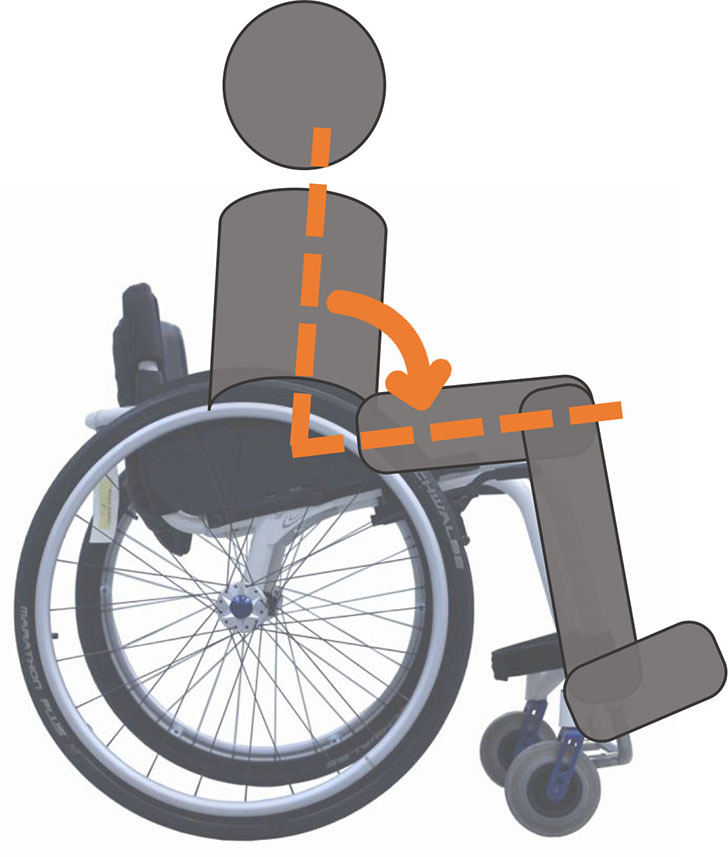	*Positional*	• Hip flexion angle •Knee flexion/extension angle
Seat height	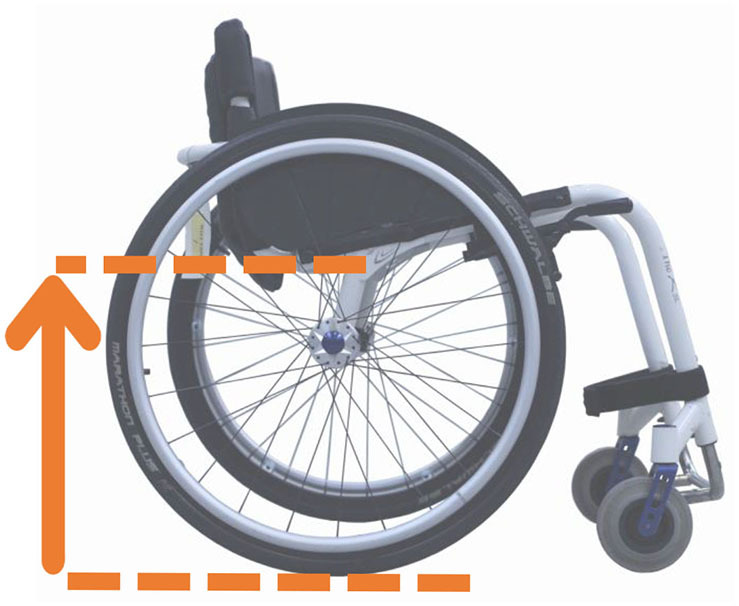	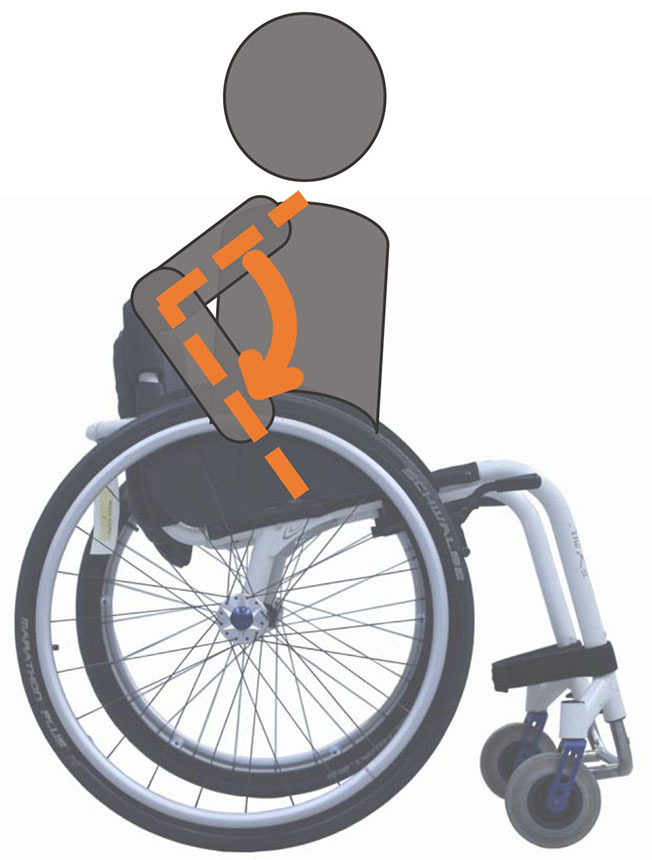	*Positional*	• Vertical distance between the floor and the back of the seat • Vertical distance between the rear-wheel axle and the back of the seat • Elbow flexion/extension angle • Elbow and shoulder flexion/extension angles • Difference in height at the top of the head • Padding thickness
Seat fore-aft position / Rear wheel axle fore-aft position	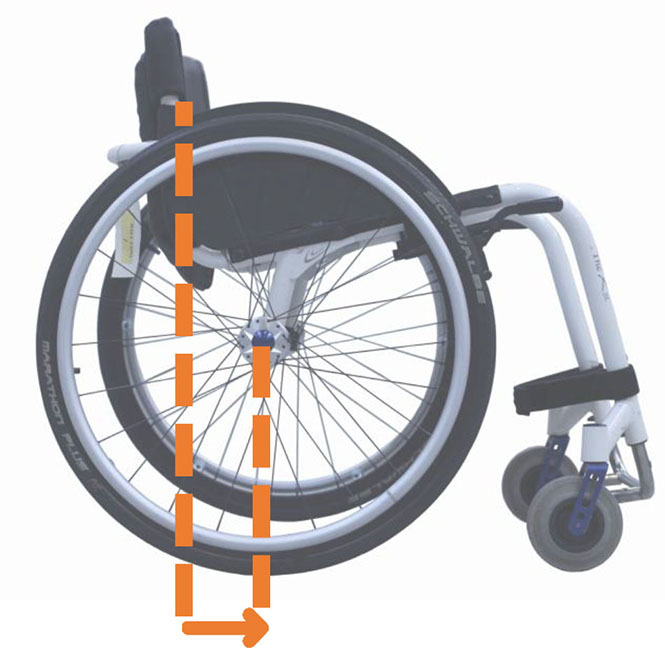	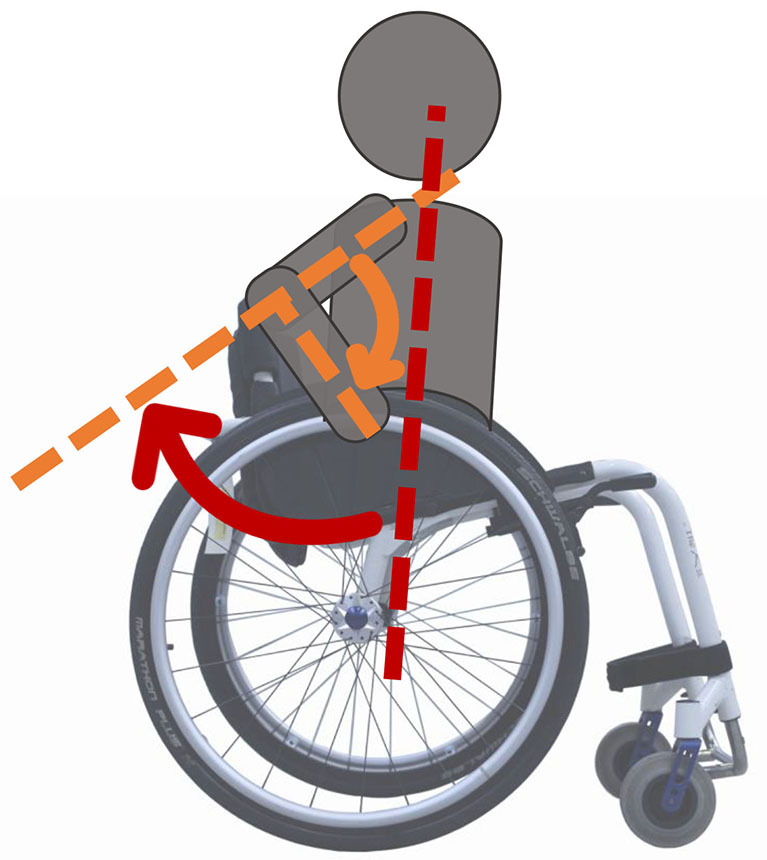	*Positional*	• Fore-aft position of the seat (Rear-wheel axle horizontal position) relative to the rear-wheel hub (resp. to the seat) (absolute or relative to anthropometric features) • Elbow and shoulder flexion/extension angles • Backrest thickness • Seat depth

* *Examples arbitrarily chosen by the authors*.

The “absolute” formalism has the merit of being self-explanatory as it echoes the measurements of manufacturers and occupational therapists. Still, some of the recommendations provided by the ISO standards are not practical to implement in a clinical or even research context (e.g., seating and wheel dimensions measured with a specific dummy in the MWC seat) and therefore are not always followed (71), leading to different measurements for the same characteristic (e.g., seat depth taken as seat upholstery depth or as the distance between the front of the seat upholstery and the intersection between the seat and the backrest).

The “relative” formalism can impose closer joint configurations between subjects, mitigating the effect of anthropometric differences and therefore of inter-individual variations on the studied outcome parameters (48), which is relevant to study the performance of the musculoskeletal system. Yet, by doing so, the absolute differences between the configurations are different for each participant, leading to non-uniform variations in the MWC mechanical parameters such as stability or rolling resistance among participants.

Both formalisms have their pros and cons, but the lack of consensus over which formalism to use with respect to the aim of the study combined with the almost systematic absence of the necessary data to switch from one formalism to the other complicates the comparison of results across articles.

Hence, the authors suggest the community provide a consensual way to describe MWC configurations, which would depend on the purpose of the study and would involve absolute or relative descriptions. Future articles are also encouraged to provide data allowing for the conversion from one formalism to the other.

### Importance of Methods and Experimental Setups

The multiplicity of methodologies used in the literature to study the effect of MCW configurations on propulsion biomechanics is explained by the fact that they each have their own advantages and drawbacks. The following paragraphs tackle the main methodological aspects:

#### Experimental Environments

When studying MWC propulsion, the first important methodological choice is the “experimental environment.” It can vary from free overground propulsion to propulsion on a wheelchair treadmill, on a roller ergometer or on a stationary wheelchair simulator. Each condition has its advantages and disadvantages, summarized in Table 3. For instance, overground propulsion appears to be the most ecologically valid testing environment, offering infinite trajectory possibilities, but it reaches its limitations when trying to monitor propulsion biomechanics and to control power output between configurations. Conversely, treadmills and roller ergometers are suitable for instrumentation, but a familiarization period is needed for the user and only straight-line propulsion can be simulated. Studies on treadmills must also prevent the subject from falling using a security system which impacts measurements.

**Table 3:** Experimental conditions advantages and disadvantages for manual wheelchair (MWC) propulsion evaluation.

**Experimental environment**	**Advantages**	**Disadvantages**
Overground	- Most ecologically valid (“realistic”) testing environment (requires trajectory and stability management while propelling) - All movements are possible - Can fit any MWC (including the user's own MWC)	- Changes are limited by the used MWC - Instrumented wheels change the MWC inertial properties, influencing propulsive torque - Difficult to control the velocity or the power output
		
Treadmill	- Can fit any MWC (including the user's own MWC) - Need to control trajectory and stability - Physiological and kinetics results close to overground propulsion - Control of speed and power-output - Effect of trunk motion taken into account	- Changes are limited by the used MWC - No acceleration or sprint testing - No turning, asymmetric propulsion - Cross-slopes conditions difficult to safely reproduce - Familiarization period needed - Security system impacts measurements
		
Roller ergometer	- Can fit any MWC (including the user's own MWC) - Physiological and kinetics results closest to overground propulsion regarding other ergometers - Control of resistance/power output with certain ergometers	- Changes are limited by the used MWC - Straight line propulsion simulation only (except for separated rollers ergometers with visual feedback) - Trunk motion has no impact on MWC velocity and stability
		
Stationary wheelchair simulator	- Easy to adapt to every participant - Any setting can be varied independently - Adjustable resistance - Can be easy to change the settings without interdependence with others setting	- Straightforward propulsion simulation only (except for haptic controlled ergometer and visual feedback) - Trunk motion has no impact on MWC velocity and stability

Comparisons of different experimental environments (e.g., overground, treadmill, roller ergometer, stationary wheelchair simulator) were previously performed by numerous authors (72–75) who agreed that the different experimental environments could be considered similar for the study of MWC propulsion. However, they only considered straight line displacement at steady state speeds. Moreover, it was already shown that MWC configuration affects the fore-aft stability during overground propulsion (16) and that roller ergometers or stationary wheelchair simulators prevent such a phenomenon from occurring. Hence, recommendations about which experimental environment should be used depending on the purpose of the study would provide more adapted results when studying the effect of MWC configuration.

#### Control of Propulsion Speed and/or Power Output

Among the articles included in this review, most do not report the actual resistance or power output. Also, many asked the participants to choose “comfort” or “self-selected” propulsion speeds without reporting the actual speed. The impossibility to assess power output prevents from making synthesis by compiling results of different studies because speed and power output can critically influence the effect of a change in MWC characteristics on propulsion biomechanics (16). Therefore, both speed and power output should always be documented. However, accurate assessment of power output is not always straightforward and special care is necessary for its quantification (9, 76).

In addition, this assessment needs to be done for every configuration tested by a participant because both resistances (rolling and steering) and MoI are affected by changes in MWC configuration. Depending on the objective of the study, it would be necessary either to maintain power output between configurations or to report the resulting change in power output due to a change in MWC configuration. Indeed, if the goal focuses on performance of the musculoskeletal system resulting from changes in joints configuration induced by a change in MWC configuration, it would be necessary to maintain the power output between configurations. Because altering the velocity is known to affect propulsion biomechanics (77, 78), the rolling resistance needs to be adapted, that is necessary for experiment with propulsion overground, on a treadmill and on roller ergometers. Through all the studies included in this systematic review, there is only one study that performed such adaptation in power output (23). However, if the objective of the study is actual displacement performance, change in power output should not be compensated for, but should still be assessed and reported.

Moreover, regarding speed, it has already been shown that participants' self-selected speed on a treadmill is lower than their speed overground (79). Also, the usual speed studied in the literature (1 m.s^−1^) is above the average daily propulsion speed of MWC users (0.5–0.8 m.s^−1^) (80), but this is a consequence of averaging speed over short displacements from standstill to full stop. Other tasks than steady-state propulsion, while more representative of daily propulsion, are however left out when studying the effect of MWC configurations, likely due to the experimental environment.

#### Measurement Systems

Along with the various experimental environments, a wide variety of measurement systems were used, from optoelectronic motion capture systems to IMUs, EMGs, pressure sheets, video cameras, force plates and instrumented wheels; each coming with its own pros and cons. For instance, IMUs allow to overcome the spatial restriction imposed by optoelectronic motion capture systems, enabling field measurements, but are less accurate to assess body orientation (81). Ideally, beyond their level of accuracy, measurement systems would not noticeably impact the subject's propulsion and the MWC characteristics. However, it is necessarily the case for some measurement systems such as instrumented wheels which modify wheel and MWC mass and mass moments of inertia (82, 83). Yet, the interest of measuring one parameter could be higher than the limitation induced by the measurement system. When using such a system, its expected impact on the results should be discussed in the study.

#### MWC Used

Another important methodological choice is the MWC used for experimentation, which can either be the participant's own MWC or the same MWC for all participants, with adaptations to each participant or not. Using the same MWC for all participants standardizes some variables and makes the experiments easier to carry out and the results easier to interpret. Using each subject's personal MWC would be more realistic but would generate variations on MWC configurations and thus on power output. In that case, a precise description of each MWC initial configuration should be provided for this choice to be relevant.

#### Participants

Choosing to study participants in their own MWC implies the recruitment of experienced MWC users for the experiments, which is generally associated with recruitment difficulties. Despite these difficulties, over 60% of the articles included in this review involved experienced MWC users (Table 1). The other articles involved novice AB subjects. The impact of studying MWC propulsion with novice AB participants has already been investigated multiple times, showing differences in power output (84), mechanical efficiency (85), energy expenditure (86), upper limb muscle recruitment (29) and kinematics (30). Based on these findings, generalization of results obtained on AB subjects to MWC users should be cautiously done. Despite numerous studies on the training of novice AB subjects (14, 87–91) showing significant improvements in propulsion technique, no article has yet been published on the amount of training necessary to achieve propulsion parameters (i.e., stroke pattern, timings, joint kinematics, forces, etc.) like those of experienced MWC users. One must be careful about the fact that the fatigue onset does not emerge at the same time for experienced and inexperienced users, and therefore propulsion time must be adapted when developing an experimental protocol. Additionally, AB subjects' morphology can be different from impaired users. However, despite these notable differences between MWC users and AB subjects, it remains possible that the conclusions on the effect of a MWC adjustment obtained in AB subjects remain valid for MWC users.

An alternative to recruiting AB subjects to compensate for the difficulty of recruiting MWC users is to enroll MWC athletes instead. Indeed, despite differences in their physical abilities, a recent study found that athletic users, that are generally easier to recruit for experiments, could be considered equivalent to non-athletic users when studying kinematic and kinetic parameters during daily propulsion (92).

It should be noted that MWC users are often considered as a homogeneous population despite being composed of a wide variety of people (spinal cord injury, multiple sclerosis, cerebral palsy, lower limb amputees, elderly people, etc.). This variability within the same group should be considered in studies, either by including diverse participants or by replicating the experiments on multiple cohorts.

#### Experimental Task

Obviously, the experimental task plays a major role in the comparison of results. Despite the recent recommendations that biomechanical research should concentrate on initiating movement, maneuvering MWC and stopping to be more representative of the actual use of a MWC in a natural environment (80, 93, 94), researchers still tend to focus on studying straight-line propulsion at steady-state speed (61% of the studies). This is less of a concern for sports-oriented studies which tend to implement multiple tasks involving different speeds in their experimental protocols. However, in the latter case, the trend of developing specific tests in each study could make comparison and literature synthesis difficult.

### Number of MWC Characteristics Investigated

The next challenge to consider is the number of MWC characteristics investigated. Because geometrical characteristics might not have independent effects on outcome parameters, conclusions drawn from experiments performed using a given initial configuration might differ when another initial configuration is used. In other words, it means that the cross-effect of geometrical characteristics should be considered and that future studies should vary multiple geometrical characteristics and interpret the results accordingly. However, as displayed in Table 4, most articles studied a single MWC characteristic and a large majority studied either one or two MWC characteristics (respectively 60% and 88% of the studies). Indeed, increasing the number of investigated characteristics impacts the number of configurations to test which could compromise results due to subject fatigue or weariness. This bias can be reduced through order randomization of the tested configurations, which most of the studies did (i.e., 88% of the studies). Additionally, one must consider the amount of time necessary for one participant to adapt to a new configuration, which also impacts the total duration of the experiment.

**Table 4:** Total number of configurations tested, and MWC characteristics investigated per article reviewed^*^.

**Total number of tested configurations**	**Number of MWC characteristics investigated**			
	**1**	**2**	**3**	**4**
1		(44)		
2	(20, 26, 27, 34, 39, 49, 57–59)	(46)		
3	(24, 25, 32, 35, 36, 38, 43, 45, 54, 55)			
4	(19, 23, 28, 41, 52, 53)	(60)		
6		(22, 31)	(56, 62)	
8	(48)		(40)	
9		(42, 47, 50, 51)		(21, 37)
12		(29, 30)		
27		(18)		

* *Articles comparing distinct MWCs rather than a single MWC with distinct settings were not included in the table (n = 2)*.

Promising techniques exist today to overcome the issue of testing multiple characteristics simultaneously such as fractional factorial experimental design or numerical simulations.

#### Factorial Experimental Design

Because the number of investigated configurations increases exponentially with the number and the range of settings under study when using full factorial experimental design, some authors proposed to use fractional factorial designs, allowing for proper extrapolation of the results from a minimal number of configurations. Two articles listed in this review (21, 37) used Taguchi's methods (95) to reduce the number of configurations to test from 81 to 9, while varying simultaneously 4 settings. It must be noted that one hypothesis of Taguchi's experimental design is that input variables should be independent or have known simultaneous effect on the outcome parameters. This hypothesis was a major concern in both articles and remains unverified.

Therefore, further studies should first consider studying setting interactions to define those that can be neglected. Then, future studies could rely on experimental design to expand knowledge on MWC.

#### Numerical Simulations

Another solution to avoid experimental limitations is to resort to numerical simulation. Some studies already embraced this approach based on simplified 2-D wheelchair propulsion models (14, 96–99), or through 3-D musculoskeletal simulations (100). Still, all these techniques rely on experimental data to feed the model.

Recently, fully-predictive simulation relying on optimal control theory was implemented to study MWC propulsion (101) and the technique was used to study the effect of seat position during sport propulsion on roller ergometer (102), drawing meaningful perspectives. Contrary to the other previous numerical techniques, fully-predictive optimal control simulation does not require experimental data. However, these simulations are still relying on simplified 2-D models due to computational cost, and their application is limited to straight line propulsion on ergometer where they represent the model that needs to be implemented.

Hence, despite the unquestionable interest of numerical techniques to limit or to dispense with subjects' participation in experiments, a substantial workload remains. In particular, further work should focus on the validation of numerical techniques and the inclusion of subjects' variability to represent the various physical capacities of MWC users.

## Limitations

Through the methodological process described in the “Methods” section, it remains possible that the current review is still not exhaustive and that some articles are missing. In particular, articles not written in English were excluded and could have brought broader knowledge. However, the authors think this would neither alter the analysis done on methodology nor the recommendations that were made for future studies.

The authors also acknowledge that the focus of the present review on MWC propulsion does not allow to draw conclusions on the effects of MWC configuration in the MWC user daily life, as stability, accessibility, compatibility with accommodation arrangement, etc. should also be considered. However, most of the recommendations made here to study propulsion would remain valid for these other aspects.

Another limitation of this review is the focus on experimental methodology, which does not include biomechanical models and data processing choices, such as angle sequences or even coordinate system in which forces and moments are expressed (103–105). Standardization efforts are also needed on these aspects.

Despite these limitations, this review provides the scientific community with perspectives to coordinate research teams especially through consensual standardization and assistance for methodological choices depending on the aim of the study.

A quality assessment of the articles was not considered relevant in this review as the goal was to identify the different methodological choices necessary to study the effect of MWC configurations on propulsion and not to evaluate results from the different articles relative to their methodologies.

## Conclusion

The 45 articles reviewed in this article were designed to understand the impact of MWC configuration on propulsion biomechanics, a goal that is still not fully accomplished today. To achieve a global understanding of the relationship between MWC configuration and propulsion biomechanics, it is crucial to evaluate the impact of each MWC characteristic, in the wider range possible, on each outcome parameter studied, and for each experimental task (e.g., straight-line propulsion, turns, curbs, slope, cross-slope). Such a huge amount of work could only be done through collaboration between research teams on a global scale. However, this work needs standardization and recommendations beforehand, to avoid the pitfalls caused by using unsuitable methodologies (mainly due to limitations of lab facilities). Indeed, because each equipment is more adapted to certain study objectives than others, future recommendations could assist researchers in adapting their research goal to their available equipment. A standardization effort in reporting MWC configuration should also be done earlier on.

## Data Availability Statement

The original contributions presented in the study are included in the article/Supplementary Material, further inquiries can be directed to the corresponding author.

## Author Contributions

CF and YP: conceptualization, methodology, screening, analysis, and writing—original draft. JB and PT: supervision and writing—review and editing. CS: conceptualization, methodology, analysis, supervision, and writing—review and editing. All authors contributed to the article and approved the submitted version.

## Funding

This study was supported by the French Ministry of Sports (grant 19r27) and was part of the Paraperf Project supported by the Program Investir l'avenir (grant ANR-19-STPH-0005).

## Conflict of Interest

The authors declare that the research was conducted in the absence of any commercial or financial relationships that could be construed as a potential conflict of interest.

## Publisher's Note

All claims expressed in this article are solely those of the authors and do not necessarily represent those of their affiliated organizations, or those of the publisher, the editors and the reviewers. Any product that may be evaluated in this article, or claim that may be made by its manufacturer, is not guaranteed or endorsed by the publisher.
