# Supplementary material for: How Was Studied the Effect of Manual Wheelchair Configuration on Propulsion Biomechanics: A Systematic Review on Methodologies
Source: Front Rehabil Sci. 2022 May 2;3:863113. doi: 10.3389/fresc.2022.863113 (PMC9397681; doi:10.3389/fresc.2022.863113)
Supplement: Supplementary file 2 [file Data_Sheet_2.PDF]

	Population									Measurements					
Article	Nb	Specificity	Title / Objectives	Experimental condition	Wheelchair used	Wheelchair reference configuration	Sports-oriented	MCW characteristic(s) studied	Range of tested MCW characteristic	Experimental device	Experimental task	Outcome parameters	Consideration of the impact on the rest of the configuration	Principal conclusion	Limits
Aissaoui et al., 2002	14	Older MWC users (mean = 68yo)	Effect of the system tilt and back recline angular variations on the biomechanical efficiency during a wheelchair propulsion cycle	Roller ergometer	Custom-built wheelchair	0° camber;<br>26.7cm handrim radius;<br>30.5cm rear wheel radius;<br>2in cushion;<br>Adjustable tension backrest	-	Seat-to-backrest angle (SBA);<br>System tilt angle (STA)	SBA: 95, 100, and 105°<br><br>STA: 0, 5, and 10°	SmartWheel;<br>Roller ergometer;<br>Strain gauge sensor	Propulsion during 1min20 at 1m.s (last 20 seconds studied)	Handrim kinetics (Fraction of Effective Force);<br>Maximal isometric forces	Slot slider mechanism to make tilt and backrest positioning independent from the horizontal and vertical position of the wheel axle with respect to the shoulder	System tilt angle but not back recline significantly affects biomechanical efficiency (=Fraction of effective force * (1-peakValue/maximal voluntary force))	MCW fixed to the ergometer: no impact of seat angle on stability
Bertolaccini et al., 2018	11	AB male	Influence of the position of the rear wheels axle (and use of accessories) on the activity of upper limb muscles during propulsion	Flat floor	Adjustable manual wheelchair with rigid frame	-	-	Seat fore-aft position	4 positions : initial position and 50 mm forward, with and without accessories (clothing shields and armrests)	T-Sens EMG;<br>Datalogger module (CAPTIV)	Straightforward 15m sprint + Slalom with decreasing distances between the cones at maximal speed	EMG activity (mV)	-	Moving the rear wheel axle forward<br>may have a positive effect on manual propulsion by decreasing the activity of upper<br>limb muscles, although this was only found with the chair in its standard configuration<br>with accessories	Few configurations;<br>Only AB participants
Cloud et al., 2017	21	SCI	Increased Seat Dump Angle in a Manual Wheelchair is Associated with Changes in Thoracolumbar Lordosis and Scapular Kinematics during Propulsion	Roller ergometer	Own MWC	Vertical backrest	-	Seat dump angle	0° (seat horizontal) and 14°	Ergometer;<br>ShapeTape ;<br>Electromagnetic system (Liberty, Pholhemus)	3 propulsion cycles	Lordosis, kyphosis, and scapulothoracic and glenohumeral kinematics	-	The full group of participants had significantly less lordosis in the 14° condition compared with the 0° condition at all propulsion events<br>For the full group of participants, no significant differences were noted in any of the 3 glenohumeral rotations between the 0° and 14° seat dump angle conditions for all events	Limited number of propulsion studied<br>Changes observed in lordosis and scapulothoracic motion between the seating conditions may represent two different strategies to the seating change;<br>Long term effect of such seat dump remain unknown;<br>Other body segment (pelvis, forearm, hand etc.) were not measured, we don't know how they adapted to the change in seat.
Costa et al., 2009	1	Elite T-52 racing wheelchair athlete	Effect of Handrim Diameter on Performance in a Paralympic Wheelchair Athlete	Athletics track	Eagle Sports Chairs athletics wheelchair, model S 606	-	Yes	Handrim diameter	Test 1 : 0.36m and 0.37m<br>Test 2 : 0.34m 0.36m and 0.37m	Video cameras (50 and 500Hz)	12*400m (with 200m head start) & 9*5min propulsion	Push frequency, push time	Not applicable	Smaller handrim diameter = longer push time and lower push frequency	Case study : only 1 subject
Cowan et al., 2009	53	(20M, 33F) Novice older MWC users	Impact of Surface Type, Wheelchair Weight, and Axle Position on Wheelchair Propulsion by Novice Older Adults	Linoleum tile hallway	TiLite folding (model X)	5.08cm foam cushion, removable plastic side guards, anti-tip devices, pneumatic rear wheels, solid 10.16 cm diameter casters, aluminum hand rims, 0° seat inclination and camber, elbow 100-120° Flexion with hands at top dead center of rear-wheel	-	Seat fore-aft position	8cm between 2 positions, original setting not specified	2 Instrumented wheels	16 propulsion at self selected speed on 4 surfaces in 4 conditions : (1) unweighted with an anterior axle position; (2) weighted with an anterior axle position; (3) unweighted with a posterior axle position; and (4) weighted with a posterior axle position	average linear velocity, push frequency, stroke length, peak resultant force and peak tangential force	Kept the other MWC characteristics constant	On all surfaces, the anterior seat condition required lower resultant and tangential forces than the posterior condition	Only study 2 extreme positions
de Groot et al., 2013	11	AB novice	Effect of wheelchair mass, tire type and tire pressure on physical strain and wheelchair propulsion technique	Level treadmill	Double Performance BV, Gouda	24in wheels, 11.5kg, 4° camber, seat angle 15° from horizontal, backrest angle 6° from vertical	-	MWC mass + tire type + tire pressure	mass: +5 and +10Kg,<br>solid vs pneumatic tires,<br>pressure : 100, 75, 50, 25%	Instrumented wheel	4min bouts of propulsion at 1.11 m/s	Push time, cycle time, push frequency, contact angle;<br>Power output, mean and peak power, FEF	-	Tire pressure : (1) low tire pressure leads to higher power output<br>(2) high tire pressure = longer cycle time and contact angle<br>Extra mass : no effect on power output or on spatiotemporal parameters<br>Tire type : Solid tires led to higher power output	Inexperienced AB users
De Witte et al., 2020	21	Elite WC basketball players<br>14 men<br>7 women<br>classification :<br>11 players < 1.5<br>10 players > 4	Effects of seat height, wheelchair mass and additional grip on a field-based wheelchair basketball mobility performance test	Wooden indoor basketball court	Own MWC	Tire pressure 7 bars	Yes	Seat height + MWC mass	1) 7.5% lower seat height;<br>2) 7.5% higher seat height;<br>3) 7.5% additional mass centrally placed at the wheel axis;<br>4) 7.5% additional mass distributed evenly at 0.3 m in front of and behind the wheel axis;<br>5) use of rubber coated gloves to increase grip on the handrim without changes to seat height or mass	Video camera	6*Wheelchair Mobility Performance test	Total performance time + task 7 and 15 times	Kept the other MWC characteristics constant	(1) a 7.5% lower seat height resulted in<br>a faster performance on the total wheelchair mobility performance test and on the combination task, and (2) 7.5% extra mass or the use of gloves did not lead to a significant change in performance time.<br>Furthermore, high and low classification players showed similar responses to the interventions	Limited number of participants<br>using the users' own MWC but no defining the configurations and assuming they are optimally tuned<br>mobility performance =/= game performance
Desroches et al., 2006	14	Older MWC users (mean = 68yo)	Effect of the system tilt and back recline angular variations on shoulder joint moment	Roller ergometer	Custom-built wheelchair	0° camber;<br>26.7cm handrim radius;<br>30.5cm rear wheel radius;<br>2in cushion;<br>Adjustable tension backrest	-	Seat-to-backrest angle (SBA);<br>System tilt angle (STA)	SBA: 95, 100, and 105°<br><br>STA: 0, 5, and 10°	SmartWheel;<br>Roller ergometer;<br>Strain gauge sensor;<br>Motion Capture System	Propulsion during 1min20 at 1m.s (20 last seconds studied)	Shoulder joint moment;<br>Push angle	Slot slider mechanism to make tilt and backrest positioning independent from the horizontal and vertical position of the wheel axle with respect to the shoulder	No difference in shoulder joint moment (mean or peak), but wheel axle position kept constant with respect to users shoulder. No influence of STA or SBA on push frequency among older people.<br>Spatiotemporal : significant effect of SBA and STA on push angle. 0 and 5° STA increased total push angle for all SBA combined. 105° SBA had a lower push angle than 100° SBA for all STA.	MCW fixed to the ergometer: no impact of seat angle on stability;<br>Propulsion pattern not studied, could have an impact on shoulder joint moments
Faupin et al., 2004	8	Male wheelchair basketball athletes	The effects of rear-wheel camber on the mechanical parameters produced during the wheelchair sprinting of wheelchair basketball athletes	Roller ergometer	Top End X-Terminator-type basketball, Invacare	13kg, 80cm long, seat inclined by 15°, backrest vertical; backrest height = 28cm;depth = 42cm; width = 39cm; rear wheel diameter = 64cm, tire pressure 8 bar	Yes	Camber	9°, 12° and 15°	Ergometer (VP100 HANDI, HANDISOFT, HEF Tecmachine, France)	3 * 8s sprints	Mean velocity per arm cycle, cycle time, push time, total power output	Only wheel camber was varied (9°, 12°, 15°). These variations caused the respective modifications of the wheelbase (68 cm, 70 cm, and 72 cm) and the seat height (28 cm, 27.3 cm, and 26.5 cm).<br>Top to top wheel distance kept constant	Increase in camber = proportional increase in power output<br>Increase in camber = increase in residual torque and decrease of mean velocity<br>Temporal parameters : time of propelling phase is the only parameter that tends to increase in proportional to increase in camber	-
Faupin et al., 2008	7	Wheelchair Basketball players	A wheelchair ergometer adaptable to the rear-wheel camber	Adjustable angle roller ergometer	Top End X-Terminator, Invacare	13kg, 80cm long, seat inclined by 15°, backrest vertical; backrest height = 28cm;depth = 42cm; width = 39cm; rear wheel diameter = 64cm, tire pressure 8 bar	Yes	Camber	9°, 12° and 15°	Adjustable angle roller ergometer	8s sprints	Velocity per propulsion cycle, cycle time, push time, acceleration/deceleration of wheels, residual torque, power output	Caster fork angle adjusted for each camber angle.<br>Only wheel camber was varied (9°, 12°, 15°). These variations caused the respective modifications of the wheelbase (68 cm, 70 cm, and 72 cm) and the seat height (28 cm, 27.3 cm, and 26.5 cm).<br>Top to top wheel distance kept constant	Spatiotemporal parameters : No significative difference between any camber angle.<br>Camber increase = increase in residual torque	-
Gorce et al., 2012	20	Male impaired experienced WC users (10), unimpaired non-experienced WC users (10)	Analyze the influence of the two main wheelchair characteristics: seat height and antero posterior axle position on joints kinematics that participate in wheelchair propulsion, in particular shoulder and wrist	Roller ergometer	Experimental wheelchair allowing adjustment of seat height and anteroposterior axle position	47 cm seat height and a 0.0 cm antero-posterior wheel position	-	Seat height;<br>Seat fore-aft position	Seat height 44, 47 and 50 cm; seat fore-aft position: −3.0, 0.0, 4.2 and 7.2 cm	Roller ergometer;<br>Modified experimental wheelchair;<br>Motion Capture system	Propulsion at self-selected speed	Joints configuration (kinematics) at handrim contact, vertex and release and over the whole movement	Other characteristics as wheel camber, seat depth, wheel size, and seat and backrest inclination were controlled and are the same during the whole experiment.	Anterior axle and low seat positions are known to be more efficient for everyday life and recreational use but both too low seat and too anterior axle positions could increase the risk to develop a shoulder or wrist injury. Large differences exist between beginners able-bodied and experts' paraplegic subjects	2 characteristics studied independently but their interaction was not explored;<br>No information on handrim forces
Guo et al., 2006	12	AB novice	Effect of handrim diameter on manual wheelchair propulsion: Mechanical energy and power flow analysis	Laboratory environment	-	-	-	Handrim diameter	0.54, 0.43, and 0.32 m	Motion Capture system;<br>instrumented wheel	5 propulsion cycle	Upper-limb kinematics, handrim kinetics (mechanical power, power flow)	Not applicable	The larger the handrim size, the greater the work done during propulsion cycle<br>Larger handrim means greater metabolic cost	Inexperienced AB users
Gutierrez et al., 2005	13	SCI adults	Effect of Fore-aft seat position on shoulder demands during MWC propulsion (part 2, EMG analysis)	Roller ergometer	2 customized Quickie GPV Sunrise (16" and 18" seat width) with custom fabricated axle plates	Align rear axle with shoulder joint center, seat height = 80° elbow extension; height of footrest and backrest adjusted to own MWC; own cushion	-	Seat fore-aft position	Seat anterior position vs seat posterior position (8 cm posterior to rear axle)	Instrumented wheel;<br>Motion Capture System;<br>EMG (right arm)	2*10s propulsion trial at self selected speed;<br>2*10 s propulsion at fast pace;<br>2*10s propulsion with 8% slope equivalent resistance	Propulsion speed, joint forces, external joint moment, joint power	-	First quantitative measures of the effect of seat fore-aft position on shoulder EMG. Seat posterior = lower activity of primary push phase muscles during fast and graded conditions, theoretically reducing risk of fatigue for pectoralis major during higher demand propulsions	seat positions always tested in the same order

	Population									Measurements					
Article	Nb	Specificity	Title / Objectives	Experimental condition	Wheelchair used	Wheelchair reference configuration	Sports-oriented	MCW characteristic(s) studied	Range of tested MCW characteristic	Experimental device	Experimental task	Outcome parameters	Consideration of the impact on the rest of the configuration	Principal conclusion	Limits
**Haydon et al., 2019**	6	Male elite WC Rugby players	Assess whether it is possible to optimize the wheelchair set-up of elite athletes at an individual level from a reduced number of test configurations using factorial design	Sports hall	Adjustable rugby wheelchair specially designed and manufactured to see the effects of different configurations	Initial set-up similar to the player's current set-up	Yes	Seat height + seat depth + seat angle + tire pressure	Increments of ± 15 mm for seat height and depth, with seat angle and tire pressure varied by ± 5° and ±15psi from the player's configuration	IMUs;<br>Video camera;<br>Laser timing gates	5 m sprint and Illinois agility tests	Contact and release angles	-	There was a potentially improved performance for increased seat height and decreased tire pressure.<br>Variations in propulsion kinematics were also evident across seat angle (SA) levels, with a trend of contact angle closer to top dead center of the wheel, and release angles further around the wheel for reduced SA compared with the current and increased settings.	there are restrictions in how accurately an adjustable wheelchair is able to replicate finer characteristics of each individual's current wheelchair;<br>No interactions studied (orthogonal design)
**Huang et al., 2013**	12	AB	Establish whether an increased camber leads to increased energy expenditure	Laboratory walkway	Quickie GP ultra light sports wheelchair	Rear wheel diameter 0.61 m, casters diameter 0.08 m, handrims diameter 0.51 m, flexible seat and back and foam cushion. Seat width 0.43 m, seat depth 0.41 m	-	Camber	0° and 15°	Instrumented wheel;<br>Motion Capture system<br>Force plates	4s propulsion on 4m (propulsion speed = 1 m/s)	Mechanical power, net power flow, joint power, kinetic friction	-	Increasing wheel camber adds stability and provides easier handling.<br>Conversely, a camber can be disadvantageous because a greater amount of energy might be required to maneuver the wheelchair. Compared to a 0° camber, power output increased by 29% with a 15° camber. The work of power flow with a wheel camber is 15% larger than that without.	Unexperienced users;<br>simplification of muscle activation and cocontraction
**Hughes et al., 1992**	15	9 AB; 6 SCI	Biomechanics of wheelchair propulsion as a function of seat position and user-to-chair interface	Wheelchair simulator data-acquisition system	-	-	-	Seat height;<br>Seat fore-aft position	Seat height : elbow flexion of 90° and 100°;<br>Fore-aft seat position: seat/backrest intersect aligned with rear wheel axle, 15% and 20% of subject's total arm length behind this axle	"wheelchair simulator data-acquisition system"	Handrim propulsion and lever propulsion, self-selected speed	joint motion, hub torque, power and speed	Handrim diameter fixed	Wheelchair propulsion biomechanics are affected by seat position, thus an optimal seat position exists.	-
**Kabra et al., 2015**	9	AB	Hand pressure during wheelchair propelling and associated rim sizes	Laboratory : 25m-long hallway with linoleum over concrete	Lomax	seat width 470 mm, seat depth 450 mm, seat height 500 mm from the ground, Rear wheel diameter 600 mm	-	Handrim diameter	46, 50, and 54 cm	Motion Capture system;<br>Novel® pressure sensors	Propulsion over 25m, self selected speed	Hand pressures;<br>Shoulder ROM (flexion/extension, adduction/abduction, rotation);<br>Elbow ROM (flexion/extension)	-	The larger handrim resulted in higher pressures and forces in the hand and in larger range of motion at the shoulder and elbow.<br>No impact on velocity	Small sample size
**Kotajarvi et al., 2004**	13	(10M 3F) wheelchair users	basic understanding of the relationship between wheelchair configuration and propulsion (effect on handrim) in real conditions with the examination of both timing and force application	Smooth level tile floor	Quickie II ultra-lightweight sport wheelchair	Rear wheel diameter 24 in. (61.0 cm), pneumatic inner tubes, handrims diameter 20 in. (50.8 cm), polyurethane caster diameter 8 in. (20.3 cm), seat width 17 in. (43.2 cm), seat depth 16 in. (40.6), and 0° camber angle, backrest and footrest similar to the subject's chair	-	Seat height;<br>Seat fore-aft position	10 cm range in seat heigh over 4 settings;<br>8 cm range in seat fore-aft position over 3 settings	Motion Capture system;<br>Instrumented wheel	Propulsion on a 20 m section at a self-selected comfortable speed	Mean Chair Speed (m/s)<br>Stroke Time (s)<br>push frequency (cycles/s)<br>Stroke Distance (m)<br>Push Time (s)<br>Recovery Time (s) and 3D positions of the markers	-	Low seat positions resulted in an improvement in studied variables. Tangential force production did not change with seat position. On the other hand, peak radial and axial handrim forces were significantly higher in the lowest seat positions. No difference in Fraction of Effective Force (FEF) with changes in seat position.	wheelchair velocity was not strictly controlled and the subjects were only instructed to try to maintain a similar velocity between trials;<br>Axle adjustment approach meant that the subjects all used the same wheelchair
**Lafta et al., 2018**	10	AB men (novice MWC users)	Impact of rear wheel axle position on upper limb kinematics and electromyography during manual wheelchair use	Laboratory: flat ground	Invacare action 2 NG	Seat height 48cm	-	Seat fore-aft position	Initial configuration +3 and +6cm	Motion Capture system;<br>trunk and shoulder EMG	5 * 5 propulsions (no start-up or stopping)	kinematic data (propulsion: peak joint angle, ROM), muscle activation signals	Seat height constant (48cm)	Forward seat position = larger upper limb ROM and higher muscular activities	Novice AB subjects (so no seat posterior positions so that they don't tip over)
**Lee et al., 2012**	20	AB adults	Effect of Wheelchair Seat Height on Shoulder and Forearm Muscle Activities during Wheelchair Propulsion on a Ramp	Slope	-	-	-	Seat height	Seat height corresponding to elbow flexion angles of 0°, 30°, 60° and 90°	EMG (pectoralis major, serratus anterior, extensor carpi radialis, flexor carpi radialis, anterior deltoid, biceps brachi, triceps and lattisimus dorsi)	3m maximal speed propulsion on ramp 1:12	Muscular activations	-	On a ramp, lower seat = more activation of pectoralis major (not the case on flat ground)<br>"Our results indicate that on a ramp, a wheelchair height eliciting an elbow joint of less/greater than 60° flexion interferes with user stability"	MWC initial configuration not described
**Lee et al., 2016**	14	AB	Effects of backrest thickness on the upper arm and trunk muscle load during wheelchair propulsion	Overground	Same for all	own configuration<br>(Lumbar pads height ~= L3)	-	Seat fore-aft position	0, 3 and 6cm thick padding	IMU attached to the upper and side trunk	30 propulsions	Single Vector Magnitude (SVM) for muscle activities (sum of the acceleration in all direction)	-	Significantly lower SVM with 3cm padding	Estimation of muscular activities using IMUs can be discussed
**Lin et al., 2020**	36	13 AB + 23 experimented users	Evaluate the relative influence of operator (shoulder position, aerobic capacity, propulsion strength) and wheelchair factors (mass, weight distribution, frictional loss) on propulsion effort during over-ground wheelchair maneuvers	tile or carpet surface	Own wheelchair (for MWC users) + random pre-configured WC (for AB)	Copy of own MWC or random configuration	-	Seat fore-aft position	-	Force gauge/dynamometer;<br>Video camera;<br>iMachine;<br>Accelerometers	figure-8 propulsion	Shoulder position;<br>Propulsion strength;<br>Deceleration;<br>Weight distribution	-	The results indicate that wheelchair configuration, (weight distribution) had a greater influence on propulsion effort than operator fitness.<br>Weight distribution can be affected by the body mass distribution and posture but is most affected by the axle position of the rear wheels. Axle position impacts shoulder position as well as the inertia and energy loss parameters of the wheelchair system.	All categories of participants mixed
**Louis et al. 2010**	20	Male impaired experienced WC users (10), unimpaired non-experienced WC users (10)	Analyze the influence of wheelchair configuration on muscle activation of prevalent actors in wheelchair propulsion.	Roller ergometer	Experimental wheelchair allowing adjustment of seat height and anteroposterior axle position	seat height 47 cm, antero-posterior wheel axle position 0cm	-	Seat height;<br>Seat fore-aft position	Seat height 44, 47 and 50 cm; seat fore-aft position: −3.0, 0.0, 4.2 and 7.2 cm	Roller ergometer;<br>Modified experimental wheelchair;<br>Motion Capture system;<br>EMG	Propulsion at self-selected speed	Muscle activation;<br>Propulsion phases	-	seat height: muscle activation increased for lower seat positions<br>fore-aft position: forward axle position increase muscle activation	-
**MacPhee et al., 2001**	10	AB novice	The effect of knee-flexion angle on wheelchair turning	Laboratory: flat painted concrete	Quickie LXI wheelchair, Sunrise	Seat depth 41cm, frame width 46cm, backrest height 37cm, caster diameter 13cm, adult size armrests, 27.5cm above seat with 25cm-long pads, rear-wheel diameter 61cm, "axle plates were adjusted to the second from the furthest back position and the middle of the three vertical possibilities"	-	Knee angle	full extension (0°) and full flexion (120°)	Video camera;<br>Stopwatch	2 * 2 angular velocity test (900° as fast as possible in the direction of non dominant arm in a 1.6m diameter circle, only 720° studied)	Time to perform task;<br>Ease of turning perception;<br>Horizontal CoM position;<br>Rolling Resistance;<br>Turning resistance;<br>Rear-wheel traction	Not applicable	With knees flexed, angular velocity was 40% faster, overall length was reduced by 39%, COM was 38% closer to the rear wheel axle, rolling resistance was 21% lower, turning resistance decreased by 17% and rear-wheel traction increased by 12%	COM and yaw moment of inertia obtained from a model, not measured; Extreme positions studied;<br>An answer to whether the advantages of the hyper flexed knee position generally outweigh the disadvantages will require further study
**Mason et al., 2011**	14	(11M 3F)<br>Highly trained wheelchair athletes<br>(11 basketball 3 tennis)	Effects of Camber on the Ergonomics of Propulsion in Wheelchair Athletes	Treadmill	Top End Transformer, Invacare	weight = 11.6 kg, wheel size = 0.635 m, tire pressure = 120 psi.<br>Minor adjustments made to copy elbow TDC angle (kept constant even when changing camber)	Yes	Camber	15°, 18°, 20° and 24°	Treadmill;<br>Video cameras	4* 4min propulsion at 2.2m/s on 0,7% gradient treadmill	push angle, hand contact, push times, shoulder flexion abduction, elbow and trunk flexion, wrist extension	With different axle, top-dead-center distance kept constant when changing camber (=48cm)<br>Seat height adjusted to elbow angle on own MWC across all camber	Increase in camber = significant increase in user power output, and significant increase in Gross mechanical efficiency (But fatigue might appear sooner)<br>Biomechanical parameters not significantly affected, but shoulder and elbow ROM in sagittal plane differed significantly	highly trained athletes;<br>Only large camber tested (>15°)
**Mason et al., 2012a**	13	Highly trained wheelchair basketball players	Determine the effects of different wheel sizes with fixed gear ratios on the maximal effort mobility performance of wheelchair basketball players during over-ground propulsion.	Sports hall	Top End Transformer, Invacare	Seat height copied from own MWC,<br>mass 11.6 kg	Yes	Camber	15 °, 18 °, 20 °, 24 °	Velocometer;<br>Timing gates	20m sprint, linear mobility and maneuverability drill	Velocity, acceleration, push frequency	Top dead center distance maintained constant (= 48cm);<br>Replicating the seat height of each participant's current sports wheelchair in the adjustable wheelchair by using the elbow angle elicited when the hands were placed on top-dead-center of the wheel, then maintaining this elbow angle between cambers by making minor adjustments to the seat height.	24° camber has negative effects on linear mobility performance, 15° camber has negative impact on maneuverability performance. 18° setting favorable for all aspects of linear and non-linear mobility performance in comparison to the other settings investigated, particularly for young or inexperienced athletes.	Participants are highly trained athletes, already used to high camber
**Mason et al., 2012b**	13	Highly trained basketball wheelchair players	Determine the effects of different wheel sizes with fixed gear ratios on the maximal effort mobility performance of wheelchair basketball players during over-ground propulsion.	Sports hall	Top End Transformer, Invacare	Seat height copied from own MWC,<br>mass 11.6 kg	Yes	Rear wheel diameter	0.59 m; 0.61 m; 0.65 m	Velocometer;<br>Timing gates	20m sprint, linear mobility and agility drill	Velocity, acceleration, push frequency	Some MWC characteristics maintained constant when changing camber	0.65 m wheels improved the maximal effort sprinting performance without negatively influencing initial acceleration or maneuverability performance.	Performance only described as time to perform the task

	Population									Measurements					
**Article**	**Nb**	**Specificity**	**Title / Objectives**	**Experimental condition**	**Wheelchair used**	**Wheelchair reference configuration**	**Sports-oriented**	**MCW characteristic(s) studied**	**Range of tested MCW characteristic**	**Experimental device**	**Experimental task**	**Outcome parameters**	**Consideration of the impact on the rest of the configuration**	**Principal conclusion**	**Limits**
**Mason et al., 2012c**	13	Highly trained basketball wheelchair players	Effects of Wheel and Hand-Rim Size on Submaximal Propulsion in Wheelchair Athletes. (Fixed gear ratio = diam roue/diam MC)	Treadmill	Top End Transformer, Invacare	Seat height copied from own MWC using elbow extension; 18° camber; 120 psi tire pressure	Yes	Rear wheel diameter	Wheel diameter: 24, 25 and 26 in.<br>Associated handrim diameter: 0.533m, 0.552 m, and 0.585 m	SmartWheel;<br>Video cameras;<br>Motion Capture System	3* 4min propulsion at 2.2m/s on 0,7% gradient slope	Kinetics (forces and moments on handrim, work per cycle, power output, resultant forces, tangential forces, fraction of effective force, rate of force development, forces and moments per strokes then averaged);<br>Temporal parameters (push time, push angle) + Kinematic data (angular displacement of all upper body segment joints)	-	Smaller wheels = increased rolling resistance = increased power output;<br>No significant effect on push frequency and push time. No significant effects on upper body joint kinematics either.<br>Work per cycle diminished as wheel size increased.<br>Smaller wheels = greater push angle.<br>Larger wheels = decrease in resultant and tangential forces on Handrim, but no significant effect on mean Fraction of Effective Force	Only one smartWheel with weight on the opposite wheel to balance: Total MWC weight = 18kg<br>Top to top rear wheel distance not maintained constant with different wheel diameter: 24 inches = 0.496 m, 25 inches = 0.477 m, and 26 inches = 0.468 m
**Masse et al., 1992**	5	Male paraplegics (racing, basket or sled hockey)	Wheelchair propulsion for various seating positions (2 heights * 3 fore-aft positions)	Roller ergometer	Specific adjustable racing wheelchair	Camber 8°, seat base 12.5°, backrest 90° from seat	-	Seat height;<br>Seat fore-aft position	Seat height: low (distal phalanges of 2nd finger aligned with lowest portion of handrims) or high (10% of subject armlength above low position)<br>Seat fore-aft position: intersection backrest/seat at 1.2, 4.4 and 7.6cm behind rear wheel axle	EMG (biceps brachii, triceps brachii, pectoralis major, deltoid anterior and posterior);<br>Video camera	6*60% of max speed over 90s (3cycles studied)	EMG;<br>Kinematic data (cycle time, pushing phase, recovery phase, pushing time, recovery time, pushing frequency and degree of contact)	For each experimental condition, wheel camber, seat base and backrest angle remained constant	Lower seat position : less EMG activity recorded, smoother upper limb motion, backward low had overall lowest IEMG and elbow and forearm acceleration slopes less abrupt.<br>Location and orientation of hand/handrim contact found to be very important: less EMG and more degrees of contact when in low positions.<br>Change in seat position did not affect trunk angular momentum	-
**Mulroy et al., 2005**	13	SCI adults	Effect of Fore-aft seat position on shoulder demands during MWC propulsion (part 1, kinetic analysis)	Roller ergometer	2 customized Quickie GPV Sunrise (16" and 18" seat width) = with custom fabricated axle plates	Align rear axle with shoulder joint center, seat height = 80° elbow extension; height of footrest and backrest adjusted to own MWC; own cushion	-	Seat fore-aft position	Seat anterior position vs seat posterior position (8 cm posterior to rear axle)	Instrumented wheel;<br>Motion Capture System;<br>EMG (right arm)	for both position:<br>2*10s propulsion trial at self selected speed<br>2*10 s propulsion at fast pace<br>2*10s propulsion with 8% slope equivalent resistance	Propulsion speed, joint forces, external joint moment, joint power	-	Moving seat posteriorly reduces superior shoulder joint force, but did not affect extension moment or sagittal power generation. Shoulder joint forces, moments and powers increased significantly during fast and graded propulsion	-
**Murata et al., 2001**	15	AB novice	The Effect of seat fore-aft/horizontal on Torque Development for Wheelchair propulsion	Custom ergometer (substitute of handrim but real wheelchair)	-	-	-	Seat height;<br>Seat fore-aft position	9 positions defined by (shoulder, elbow angles) (50,90)(60,95)(70,100)(40,80)(50,85)(60,90)(30,65)(40,70)(50,75)	EMG;<br>Handrim kinetics	Maximum isometric exercise for 6s with hands at top dead center of rear wheels	Peak and average torque	-	Peak torque was higher for forward positions (+14.8% for forward low position) and decreased for backward positions of the handrims (minus 1.3 – 4.9%)	Hands at top dead center of the rear wheels does not represent start of propulsion !
**Rudins et al., 1997**	10	AB novice	Kinematics of the Elbow During Wheelchair Propulsion: A Comparison of Two Wheelchairs and Two Stroking Techniques	Custom-made roller ergometer	Standard vs lightweight	Lightweight : 0° camber, lowered seat height by 1 increment on axle plate, axle 3.5cm ahead of backrest, seat-to-handrim vertical distance = 7.5 for standard MWC and 13cm for lightweight MWC, pneumatic tire inflated to manufacturer's specifications 65psi	-	all<br>(comparison of 2 MWC)	-	Video cameras;<br>Reflective markers	Propulsion at comfort speed on roller ergometer with 2 MWC and 2 stroke pattern	Upper-limb kinematics;<br>Contact and release angles	-	Minimal kinematic differences;<br>Standard wheelchair appeared to have less rolling resistance on the rollers than the lightweight wheelchair.	Too many different variables: " Whether this apparent difference in rolling resistance was due to the specific rubber compound in the tires, the tread, the wheel camber, or to the inherent friction at the wheel axles themselves is unclear."
**Samuelsson et al. 2004**	12	(10M, 2F)<br>SCI thoracic or lumbar	Analyzes the effects of rear-wheel position on wheelchair propulsion and seating aspects	Treadmill and computer work situation (static)	Adjustable XLT Power, INVACARE	Backrest angle adjusted to fit each individual. Three seat widths were used to fit the wheelchair to each subject. Subjects used their own seat cushion in both seat positions.	-	Rear-wheel axle position and seat angle	Seat angles 5° and 12°;<br>Between seat positions I and II:<br>Vertical distance = 55 mm;<br>Horizontal distance is 12 mm	Treadmill;<br>External force acting via a pulley system and rolling resistance measured with force gauge;<br>Video inclinometer	Propel at 1 m/s (or adapted speed if not ok for the participant) for 6 min	Push frequency and stroke angle (from videotape), self-evaluation of comfort, Freely chosen push frequency/min, Power output, drag force, pelvic position (static)	The impact of modifying seat inclination and wheel axle position on the other characteristics were monitored. Only backrest angle changed between both positions and this change was quantified. (mean backrest angle change between positions I and II was 5.3° ± 4.0°)	No significant difference on estimated propulsion qualities between the two positions. A simple correlation between wheelchair ergonomics and propulsion efficiency may not exist. The key to a successful prescription is probably to fully understand the relationship between the user, the equipment, and the environment, including the different kinds of activities the user engages in.	-
**Silva et al. 2019**	31	AB	Evaluation of two wheelchair hand rim models: contact pressure distribution in straight line and curve trajectories	Laboratory: overground flat floor	Ortobras Star Lite	Seat width 400mm, seat depth 400mm, backrest height 400mm, Tire type and tire pressure provided	-	Handrim shape	1 - conventional handrim (metal 20mm diameter)<br>2 - Contoured handrim (polymer)	Video camera;<br>Grip VersaTek Wireless System (Tekscan Inc.)	Figure-8 propulsion at comfort speed	Hand pressure data (peaks and conditional means)	Tire type and pressure changed between configurations	Contoured handrim design was related to reduced levels of contact pressure on most hand regions, however it concentrated a high level of pressure on the medial phalanges<br>Authors currently do not recommend the feature	Tire type and pressure changed between both handrims (pressure from 36psi (conventional handrim) to 110psi (contoured handrim))
**Tsai et al., 2012**	12	AB inexperienced users	The effects of rear-wheel camber on the kinematics of upper extremity during wheelchair propulsion	A paved granular canvas to simulate outdoor wheelchair propulsion	Quickie GP ultralight (with Custom design camber plate)	-	-	Camber	0°, 9° and 15°	Motion Capture System;<br>Instrumented wheel	4 propulsions (1 m/s)<br>4-meter pathway in 4 seconds	peak joint angle, joint ROM and propulsion pattern	Rear wheels top dead center distance kept constant (40% of arm span)<br>Change in camber = change in seat height and tilt angle, not adjusted because relatively small	increase in camber = significant increase in average acceleration and maximum end angle and forward trunk flexion<br>Camber = more subjects using single loop pattern<br>No significant difference in shoulder ROM and peak angle	Only right side of the participants was studied;<br>Inexperienced users;<br>small number of subjects;<br>Small Motion Capture system area, not the best to study MWC propulsion
**Usma-Alvarez et al., 2014**	5	4 Australian wheelchair rugby Paralympians and 1 National League athlete	Identify the contribution of specific wheelchair design parameters to the performance of individual wheelchair-athlete systems, Narrow high performance wheelchair designs for the individual athlete according to performance data and ergonomic input, Develop a systematic and evidence-based wheelchair design methodology to assist the improvement of elite athletes performance.	Roller ergometer	Adjustable frame wheelchair	Level 1 (out of 3) of Taguchi's method correspond to the participant's own MWC configuration	Yes	seat height, camber, and seat fore-aft position, wheel diameter, seat and backrest angles, footplate height, width of the seat and back, wheel distance at top dead center, wheel diameters, cushion thickness, and straps requirements (fitted to each athlete)	Initial setting + 2 levels : camber -2°, + 2°, seat height +30mm & + 60mm, seat depth -30mm & +30mm	Roller ergometer	five repetitive 14 m-sprint tests in each of the nine experiments according to the standard L9 (Taguchi)	Values of (tp), (tr), and (vp) from the velocity curve directly, mean acceleration was obtained from vp, cumulative velocity (vc) vc = vpo=vp, vpo the initial velocity for each of the nine pushes for each repeated experiment + comfort assessment in a scale of low, medium, and high.	-	Is highly recommended that this method be used in conjunction with specialist knowledge of biomechanics of wheelchair propulsion and the effect of setting specific performance targets on the athlete's prolonged physical capacity as the predefined function for obtaining high performance output, could be associated with strenuous physical activity and risk of injury for prolonged time periods or at maximum exertion of physical activity.	Performance-oriented analysis, injury risk and fatigue might be interesting to consider;<br>Some unknown MWC characteristics interactions prevent from interpreting part of the results;<br>Important concepts not addressed in this article were examinations of athlete's energy consumption or joint kinematics during wheelchair propulsion.
**Van der Linden et al. 1996**	6	AB	The Effect of Wheelchair Handrim Tube Diameter on Propulsion Efficiency and Force Application	Stationary computer controlled wheelchair ergometer	Custom-built wheelchair ergometer	Handrim diameter 26cm;<br>13° camber;<br>0° seat angle;<br>90° backrest angle;<br>120° elbow flexion (seat height)	-	Handrim shape	1 - oval shaped section (25*30mm)<br>2 - cylindrical shaped section (18mm diameter)	Custom-built ergometer;<br>Video cameras	2*submaximal exercise tests	Total Net Power Output;<br>Total force applied onto the handrim;<br>Effective force;<br>Fraction of effective force;<br>Spatiotemporal propulsion parameters (cycle time, push frequency, push angle)	Not applicable	No significant effect on spatiotemporal propulsion parameters (cycle time, push angle, push frequency);<br>No significant difference in peak power output	Novice subjects might not apply realistic handrim forces
**van der Slikke et al. 2018**	20	Elite wheelchair basketball athletes<br>Classification:<br>10 high (4-4.5);<br>10 low (1-1.5)<br>7 international level,<br>13 Dutch national	Provide quantitative insight in the effect of seat height, mass and grip on wheelchair mobility performance for athletes of low and high classification.	Sports hall	-	-	Yes	seat height (+mass and grip)	7.5% lowered seat height (L);<br>7.5% elevated seat height (H);<br>(+ changes in mass and grip)	"wheelchair mobility performance monitor" (3-inertial sensor based method developed in a previous study by the author)	six ~7-minute tests with 15-30 min rest in between (with straight sprint, 360° curve, turning on the spot and combined actions)	The 6 Wheelchair Mobility Performance (WMP) outcomes : average forward speed; average speed in the best two runs (speed sections); average acceleration in the first 2m from standstill; average rotational speed in a curve; average rotational speed in the best two turns (rotation sections); average absolute rotational acceleration.	Wheelchairs characteristics, especially adjusted seat height, were altered while preserving other chair ratios. So, with elevating or lowering the seat, the height of the backrest and footplate was changed equally.	Seat height affected outcomes of both forward and rotational movement. The classification of an athlete does not seem to cause different effects on wheelchair mobility performance.	Given the heterogeneity of the group of wheelchair athletes, it is uncertain if a valid regression equation could be established, even if performance is measured in more conditions.<br>Enough time in-between tests was allowed for full physical recovery, but not for full physical or coordinative adaptation
**van der Woude et al. 1989**	9	AB male	Combined physiological and kinesiological analysis of effects of four different seat heights, during handrim wheelchair propulsion, at four different velocities.	Treadmill	solid-frame basketball wheelchair (Morien Tornado)	weight-14.5 kg;<br>hard caster diameter 0.08 m ;<br>rear wheel diameter 0.61m;<br>tire pressure 3.10^5Pa	Yes	Seat height	seat heights of 100, 120, 140, and 160° elbow extension (180° = full extension)	motor-driven treadmill (treadmill + load imposed on the wheelchair-user combination through rolling drag, and an additional external force acting via a pulley system on the wheelchair)	Each test consisted of four 3-minute exercise blocks at speeds of respectively 0.55, 0.83, 1 .11, and 1.39 $m.s^{-1}$ (2-5 $km.hr^{-1}$)	Power output, cycle frequency, work, mechanical efficiency	-	The recovery time did not show a significant trend with seat height position.<br>Seat height adjustment is critical, and related to anthropometric dimensions. Optimum seat height in terms of cardiorespiratory responses is near 100 to 120 degrees elbow angle for daily-use and basketball wheelchairs.	Lower and smaller intervals of seat height would be interesting;<br>Parameters missing : the fore-aft position of the seat, the width of the wheels, and the angulation of seat and backrest.<br>Do not neglect effect of trunk.

	Population									Measurements					
**Article**	**Nb**	**Specificity**	**Title / Objectives**	**Experimental condition**	**Wheelchair used**	**Wheelchair reference configuration**	**Sports-oriented**	**MCW characteristic(s) studied**	**Range of tested MCW characteristic**	**Experimental device**	**Experimental task**	**Outcome parameters**	**Consideration of the impact on the rest of the configuration**	**Principal conclusion**	**Limits**
**van der Woude et al. 2009**	12	(5F, 7M)<br>Recent SCI	To evaluate the effects of wheelchair seat height on wheeling efficiency and technique during rehabilitation in subjects with a spinal cord injury.	Computer-controlled ergometer	-	Camber 4°;<br>Seat angle 5°;<br>backrest angle 10°;<br>Rear wheel diameter 0,62m;<br>Handrim diameter 0,52m;<br>Handrim tube diameter 0,03m;<br>Seat fore aft position fixed individually with the subject's acromion vertically above the wheel axle;<br>Distance between the wheels proportional to bi-acromial width.	-	Seat height	8 positions/subjects : shoulder flexion from 70° to 140° every 10° (180°=full extension)	Computer controlled WC ergometer	Submaximal propulsion for each position (4min + 5min rest)<br>Exercise intensity, slope and speed, were kept constant for each subject over the 8 different exercise bouts (chosen at the beginning of the exp for each subject relatively to its capacities)	Handrim kinetics	Over the different seat height conditions, individual ergometer characteristics other than seat height were kept constant.	Based on cardio-respiratory parameters and mechanical efficiency, subjects with SCI during rehabilitation showed an optimal seat height over a range of 100–130° elbow angle during submaximal hand rim wheelchair propulsion.	-
**Vanlandewijck et al., 2011**	15	AB male students (recreational WC basket or tennis)	Describe the impact of two different deep seating positions on active trunk range of movement relative to the handrim during wheelchair acceleration from standstill; and to determine whether either of the deep seating positions adversely affects wheelchair acceleration from standstill.	Roller ergometer + flywheel	Quicky GPV handrim propulsion basketball wheelchair	seat width 0.42 m;<br>seat depth 0.55 m;<br>backrest height 0.36 m;<br>seat inclination 0°;<br>wheel diameter 0.70 m;<br>camber 6°	Yes	"seating position" (=footrest height + seat dump angle)	Hip flexion of 90°, 45° and 0°	Video camera	2 maximum effort sprints (20 sec at max speed) for each configuration	Trunk position, push characteristics, wheel displacement, speed and acceleration	-	45° of hips flexion lowers trunk ROM of start up only, and propulsion performances are not impacted.<br>90° of hip flexion has an overall negative impact (trunk ROM, acceleration during the first propulsions)<br>So during classification, athletes should be judged on reach tasks, trunk muscular activity and trunk/arm coordination	Pelvis inclination not monitored
**Veeger et al., 1989**	8	AB	Establish whether increased camber leads to a more efficient level of wheelchair propulsion in terms of physiological and movement pattern parameters.	Treadmill	Morrien-Tornado basketball wheelchair (weight 14 .5 kg, rim diameter 0.52 m, tires Vredestein DOETO)	Seat height standardized at 120° elbow angle	Yes	Camber	0, 3, 6, and 9 degrees	Video cameras	four 12-minute wheelchair exercise tests with sped increasing every three min	Kinematics	Seat height standardized at 120° elbow angle across different camber angles	No kinematic advantages were found with higher camber, but better lateral stability, lower rolling resistance, a lower downward turning moment on lateral slopes, and, in turns at higher speeds, there is less stress on the bearings.	-
**Veeger et al., 2017**	60	(44M, 16F)<br>basketball players<br>Classification:<br>20 <2,5<br>40 >=3	Improving mobility performance in wheelchair basketball	Sports hall	own MWC	own configuration + tires minimal pressure = 7 bars	Yes	all:<br>33 variables describing MWC (19), athlete (10), athlete/MWC interface (4)	Camber from 15 to 21°	Video cameras	Wheelchair Mobility Performance (WMP) test	total time + times for 12m sprint, 12m sprints with stops, rotation and rotation with stops	-	Handrim diameter and wheel axis height are predictors of mobility: increase in hand rim diameter or wheel axis height = better performance on 12m sprint with and without stops respectively.<br>Increase in mobility among less impaired players by increasing the rear seat height and decreasing the front seat height.<br>Maximal isometric force was found to be a good predictor for mobility performance in the following tests : 12m sprints, rotation and total WMP test	-
**Wieczorek et al., 2019**	1	AB novice	Effects of seat angle on the changes in the position of the center of gravity of the human body in dynamic condition	Custom-made roller ergometer	multi-gear transmission wheelchair	-	-	Tilt-angle	0°, 1.5°, 5.4°	Custom-built ergometer recording CoM during propulsion	27 * 30 propulsions at constant frequency (3 frequencies * 3 tilt angles * 3 gear ratios)	Center of Mass position	Tilt angle : all the MWC was tilted, not only the seat	Custom-built ergometer allows for the monitoring of the CoM	Tilt angle =/= seat angle in this case, all the MWC was tilted on the ergometer; only 1 subjects
**Yang et al. 2012**	36	(26M, 10F)<br>SCI from T8 to L5	Effect of Backrest Height on Wheelchair Propulsion Biomechanics for Level and Uphill Conditions	Treadmill	2 lightweight manual wheelchairs: Karma KM-8520 (16 and 18in seat width)	-	-	Backrest height	(1) high backrest as a fixed 40.6cm (16in)<br>(2) low backrest as 50% of the participant's trunk length	SmartWheels;<br>Motion Capture System	4 * 30-second propulsion trials<br>(2 different slope conditions x 2 different backrest heights) at 0,9m/s	Kinematic data, propulsion kinetic data, forces and moments on each wheel.<br>Computed : Mechanical effective force;<br>The peak extension angle and the range of shoulder flexion/<br>extension motion during the push phase of each stroke were selected for analysis	Not applicable	Low backrest: longer push times, larger stroke angles, larger shoulder extension angles at the beginning of push phase<br>3° slope: increased cadence, handrim hand contact shifted forward, larger resultant force, tangential force and propulsion torque.<br>No effect of backrest height on propulsion kinetics was found	Only low paraplegia users; higher backrest could be needed for adequate postural support
